# Compositions of carbonaceous-type asteroidal cores in the early solar system

**DOI:** 10.1126/sciadv.abo5781

**Published:** 2022-09-16

**Authors:** Bidong Zhang, Nancy L. Chabot, Alan E. Rubin

**Affiliations:** ^1^ Department of Earth, Planetary and Space Sciences, University California, Los Angeles, CA 90095-1567, USA.; ^2^ Johns Hopkins University Applied Physics Laboratory, Laurel, MD 20723, USA.; ^3^ Maine Mineral and Gem Museum, 99 Main Street, P.O. Box 500, Bethel, ME 04217, USA.

## Abstract

The parent cores of iron meteorites belong to the earliest accreted bodies in the solar system. These cores formed in two isotopically distinct reservoirs: noncarbonaceous (NC) type and carbonaceous (CC) type in the inner and outer solar system, respectively. We measured elemental compositions of CC-iron groups and used fractional crystallization modeling to reconstruct the bulk compositions and crystallization processes of their parent asteroidal cores. We found generally lower S and higher P in CC-iron cores than in NC-iron cores and higher HSE (highly siderophile element) abundances in some CC-iron cores than in NC-iron cores. We suggest that the different HSE abundances among the CC-iron cores are related to the spatial distribution of refractory metal nugget–bearing calcium aluminum–rich inclusions (CAIs) in the protoplanetary disk. CAIs may have been transported to the outer solar system and distributed heterogeneously within the first million years of solar system history.

## Teaser

Higher siderophile abundances in outer solar system asteroidal cores indicate heterogeneity between compositional reservoirs.

## INTRODUCTION

Most meteorites can be divided into two suites—carbonaceous (CC) and noncarbonaceous (NC) types—based on their distinct N, O, Ti, Cr, Mo, Ru, Ni, and W isotopic compositions (*1*–*10*). This isotopic dichotomy revealed by nucleosynthetic anomalies (especially for Mo isotopes) shows that CC meteorites are enriched in rapid neutron capture process (*r*-process) nuclides over NC meteorites; NC and CC meteorites plot along two parallel slow neutron capture process (*s*-process) mixing lines on the Mo-isotopic diagram (*11*). The dichotomy indicates that the vast majority of meteorites originate from one of the two reservoirs: NC (inner solar system) and CC (outer solar system) (*1*, *12*). The two reservoirs were likely separated by the formation of Jupiter <1 million years (Ma) after the formation of calcium aluminum–rich inclusions (CAIs) (*1*, *5*). The CC-NC dichotomy suggests that the two reservoirs did not undergo substantial mixing during the period of accretion of meteorite parent bodies (*11*).

Iron meteorites can be classified into two broad categories, “magmatic” and “nonmagmatic.” Magmatic iron meteorites show compositional evidence for having formed by fractional crystallization within well-mixed molten metallic cores in differentiated asteroids (*13*–*15*). The parent bodies of magmatic iron meteorites are the earliest differentiated asteroids, accreting within ~1 Ma of solar system history (*5*, *16*). Notably, the iron meteorite parent bodies formed earlier than the (unmelted) chondritic asteroids (*17*). Thus, magmatic iron meteorites preserve, to an extent, the chemical and evolutionary signatures of the early solar system. These signatures include the compositions and redox conditions of planetesimals, the distribution and behavior of trace elements, the sizes and numbers of planetesimals, core formation processes, and impact events.

Many aspects of the chemical signatures and planetary evolutionary histories of asteroidal cores can be reconstructed by fractional crystallization modeling (*13*, *18*–*20*). Such modeling has been performed for most magmatic iron meteorite groups: (i) the CC-iron groups IIC (*21*), IID (*22*, *23*), IIF (*24*), IIIF (*23*), and IVB (*25*–*27*), and one grouplet, the South Byron Trio (SBT) (*28*); and (ii) the NC-iron groups IC (*29*), IIAB (*23*, *26*, *30*), IIIAB (*23*, *31*, *32*), and IVA (*26*, *33*–*35*). More comprehensive modeling of group IIIF suggests that these irons might not represent an iron meteorite group formed by straightforward fractional crystallization processes (*36*).

Prior crystallization models for CC-iron groups were based mainly on a limited number of elements, such as Re, Os, Ir, Ru, Pt, and Pd (*21*, *23*, *24*, *28*) or Ga, Ge, Ir, and Au (*22*, *26*, *30*, *31*, *33*). In addition, some prior studies (*22*, *25*–*27*, *30*, *31*, *33*) need revisiting in light of new experimentally determined partitioning parameterizations (*37*).

In the present study, we use new high-precision NAA (neutron activation analysis) data, supplemented by ICP-MS (inductively coupled plasma mass spectrometry) data from the literature (*21*, *23*, *24*, *27*, *28*) for elements not measured by NAA. The recent experimentally determined partitioning parameterizations (*37*) are used along with a revised fractional crystallization model (*32*) to investigate all CC-iron magmatic groups (IIC, IID, IIF, and IVB) and the SBT. We present fractional crystallization modeling for 19 elements, applying the same modeling approach to an extensive set of elements in each group to enable comparisons among asteroidal cores. This study aims to (i) estimate the bulk compositions of CC-iron cores, (ii) explore any chemical heterogeneities observed among CC and NC cores and identify potential mechanisms that may have produced such heterogeneities, (iii) investigate the processes responsible for fractionating siderophile elements among the cores, and (iv) reconstruct the crystallization processes of CC-iron cores.

## RESULTS

Members of a magmatic iron meteorite group are products of the fractional crystallization of a single metallic core, and variations in the concentrations of siderophile elements can be modeled using a fractional crystallization approach (*13*). Sulfur and P concentrations, increasing over the course of crystallization in metallic melts, affect the behavior of siderophiles (*19*, *38*–*40*). While P concentrations can be measured accurately in most iron meteorites, any S present in the molten metallic core is largely excluded from the solid metal that crystallizes and, hence, cannot be determined directly by iron meteorite measurements. Instead, iterative modeling of the siderophile element trends in an iron meteorite group is undertaken using different initial S contents to constrain the bulk S content of the core. The distribution coefficients of siderophiles for different S and P concentrations can be calculated using the parameterizations produced from experimental determinations of their partitioning behavior (*20*, *37*). With these parameterizations, the small-step batch crystallization modeling can simulate the fractional crystallization process of metallic melts, enabling the examination of compositional effects due to equilibrium mixing of solids and liquids during the crystallization process (*31*). The formation of trapped melt and its role in the crystallization of iron cores can be assessed using trapped melt fractional crystallization modeling (*32*). In a successful fractional crystallization model of an iron meteorite group, bulk concentrations of S, P, and siderophile elements should generate fractional crystallization tracks that reasonably predict all interelement trends observed within a group.

The modeling method and details are discussed in the Supplementary Materials. The mean compositions of irons used in this study are listed in table S1; NAA replicates are listed in table S2. The bulk compositions determined as the best fit for each of the models are shown in table S3. A summary of bulk HSE (highly siderophile element) and Ni compositions from this study and the literature appears in table S4. In the next sections, we discuss the detailed modeling of each CC-iron core; Table 1 summarizes the results. For each group below, we show modeling results of Co, Ga, Ir, and Au versus As, because these four elements have distinct chemical behaviors during fractional crystallization of metallic melts (*37*), and Co, Ga, and Ir are key elements used in the classification of iron meteorites (*41*, *42*).

**Table 1.** Bulk S, P, and Ni contents; crystallization sequences; and fractions of trapped melt of CC-type iron meteorite groups compared to those in the literature. Data or information is from this study unless otherwise noted.

**Group**	**Bulk S (wt %)**	**Bulk P (wt %)**	**Bulk Ni (mg/g)**	Crystallization sequence* (%)	**Trapped melt fraction (%)**
IIC	8^†^	2^†^	110^§^	10–26^†^	–
	6 ± 2	2.2 ± 0.3	100	≤30	<10
IID	0.7^‡^	1.4^‡^	105^‡^	0–73^‡^	<14^‡^
	10^§^	1^§^	100^§^	~20^§^	<90^§^
	0.5 ± 0.5	1.9 ± 0.1	108	≤84	<15
IIF	11–13^‖^	0.4–0.5^‖^	110^§^	–	–
	5 ± 1	0.65 ± 0.05	119	≤61	<10
IVB	~2^¶^	0.65^¶^	190^§^	–	–
	~0^#^	0.4^#^	–	17–86^#^	0^#^
	1 ± 1**	–	–	–	–
	0.5 ± 0.5	0.45 ± 0.02	178	≤78	<15
SBT	7^††^	1^††^	190^§^	1–42^††^	–
	8 ± 2	1.5 ± 0.3	180	≤37	<5

*Crystallization sequences from this study all start from 0% solid to 100% liquid.

†(*21*).

‡(*22*).

§(*23*).

‖(*24*).

¶(*27*).

#(*25*).

**(*26*).

††(*43*).

### Group IIC

**Fig. 1.**
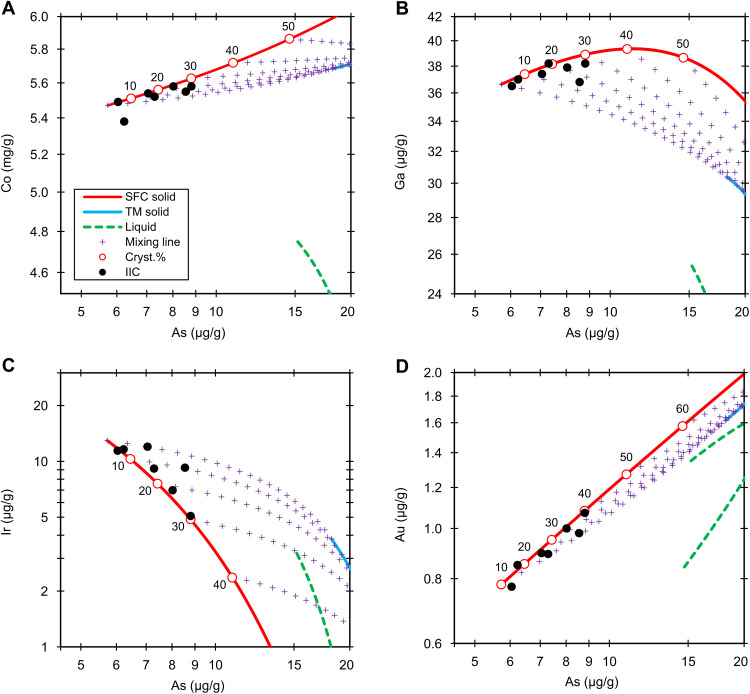
Group IIC. Fractional crystallization model (6 wt % S and 2.2 wt % P) of the Co (**A**), Ga (**B**), Ir (**C**), and Au (**D**) versus As in group IIC. The black dots are the NAA data. The red lines, blue lines, and green dashed lines denote the solid derived from simple fractional crystallization (SFC solid), solid from trapped melt (TM solid), and liquid (liquid), respectively. The purple crosses are the mixing lines (mixing line) between fractional crystallization and trapped melt solids at an increment of 5%. The labeled circles on the red lines represent the crystallization sequence (cryst.%).

We determined an initial bulk composition with 6 weight % (wt %) S and 2.2 wt % P to fit most of the 18 interelement trends. Bulk S contents were varied by 2% above and below the optimal 6 wt % S to get similar fits for all elements. We conclude that the group has initial bulk concentrations of 6 ± 2 wt % S and 2.2 ± 0.3 wt % P. The result for group IIC is consistent with the concentrations of 8 wt % S and 2 wt % P determined by modeling HSEs (*21*). Our model generally works for all elements and works especially well for P, Co, Ga, Ge, Sb, Pd, Re, Os, Ir, Pt, and Au versus As; other trends show slight scatter (Fig. 1 and fig. S2). The scatter of the Ni, W, Mo, and Rh versus As trends does not seem to have been caused by the formation of trapped melt or S variations. The scatter for Mo and Rh can be explained solely by analytical uncertainties (10 to 50%) by laser ablation (LA)–ICP–MS (*21*). For W, the relative 95% confidence limits of INAA (instrumental NAA) are 7 to 10%, which largely account for the scatter and the less-than-perfect fit of the element to the model.

The low analytical uncertainties (1.5 to 3%) and high distribution coefficient of Ir are suitable for estimating the fraction of trapped melt and the crystallization sequence of the core (*22*, *30*, *31*). The IIC irons closely adhere to the solid track with low amounts (<10%) of trapped melt. The eight IIC members represent ≤30% crystallization products of the core; the previous estimate is 11 to 26% (*21*).

### Group IID

**Fig. 2.**
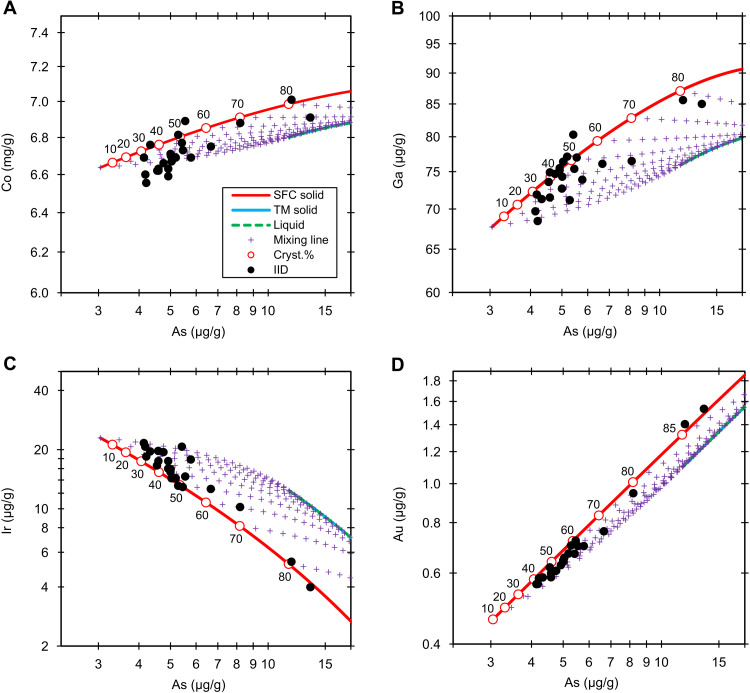
Group IID. Fractional crystallization model (0.01 wt % S and 1.9 wt % P) of the Co (**A**), Ga (**B**), Ir (**C**), and Au (**D**) versus As in group IID. The black dots are the NAA data. The red lines, blue lines, and green dashed lines denote the solid derived from simple fractional crystallization (SFC solid), solid from trapped melt (TM solid), and liquid (liquid), respectively. The purple crosses are the mixing lines (mixing line) between fractional crystallization and trapped melt solids at an increment of 5%. The labeled circles on the red lines represent the crystallization sequence (cryst.%). The liquid and trapped melt tracks overlap due to the S-free model conditions.

The element versus As trends for 15 elements in group IID can be best fitted by a bulk composition of ~0 wt % S and 1.9 wt % P. By using the bracketing method, the bulk concentrations are estimated to be 0.5 ± 0.5 wt % S and 1.9 ± 0.1 wt % P. These S and P contents are consistent with the previous report of 0.7 wt % S and 1.4 wt % P based on the Ir versus As/Au trend (*22*) but contrast with 10 wt % S and 1 wt % P based on HSEs (*23*). A more detailed comparison of the models is made in the Supplementary Materials and shown in fig. S1. Our model works well for P, Co, Ni, Cu, Ga, Ge, Ir, Sb, Pd, Re, Os, and Au (Fig. 2 and fig. S3). Although W, Pt, and Ru versus As trends are relatively scattered in the less-evolved irons, the overall trends reasonably fit these elements. Just as in group IIC, the scatter of W in group IID is also observed and should be attributed mainly to the relatively high analytical uncertainties of INAA for this element. The model works equally well for most elements despite the slight scatter of Ge and Sb versus As trends. The current collection of IID irons represents ≤84% crystallization of the parent melt. Most IID irons indicate a relatively low fraction (<15%) of trapped melt, and the maximum fraction of trapped melt in Richa goes up to 25%. The low inferred amounts of trapped melt in IID were almost identical to the previous estimate of <14% (*22*).

### Group IIF

On the basis of our new INAA analyses, we suggest that Monahans (1938), Purmela, and Corowa should be reclassified as IIF-an and excluded from the modeling. Monahans (1938) and Purmela have similar elemental concentrations and do not follow the Co, Ni, Ga, W, and Ir versus As trends of other IIF irons. Corowa has a notably lower Co concentration (6.29 μg/g) than other IIF irons (mean = 6.84 ± 0.06 μg/g); it is also lower than what the model tracks predict (>7 μg/g). Corowa roughly coheres to the IIF interelement trends, but the model cannot account for the Ru, Pt, Ir, Re, and Os versus As trends using a single S content. Hence, we tentatively classify Corowa as IIF-an. The inclusion or exclusion of Corowa, as a highly evolved iron, in the model does not significantly change the resulting bulk composition of the core. We suggest the ungrouped iron meteorite Northwest Africa (NWA) 6932 be reclassified as IIF based on the INAA analysis. The majority of Ru, Os, and Pt data and all Mo, Rh, and Pd data are from ICP-MS analysis in the literature (*24*).

**Fig. 3.**
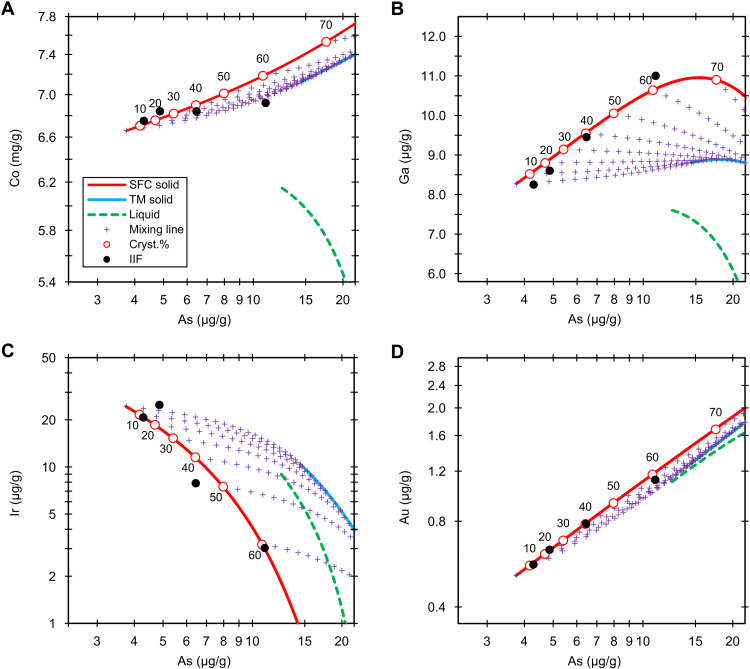
Group IIF. Fractional crystallization model (5 wt % S and 0.7 wt % P) of the Co (**A**), Ga (**B**), Ir (**C**), and Au (**D**) versus As in group IIF. The black dots are the NAA data. The red lines, blue lines, and green dashed lines denote the solid derived from simple fractional crystallization (SFC solid), solid from trapped melt (TM solid), and liquid (liquid), respectively. The purple crosses are the mixing lines (mixing line) between fractional crystallization and trapped melt solids at an increment of 5%. The labeled circles on the red lines represent the crystallization sequence (cryst.%).

The remaining four IIF irons (Del Rio, Dorofeevka, NWA 6932, and Repeev Khutor) can be best fitted by 5 wt % S and 0.70 wt % P (Fig. 3 and fig. S4). The element versus As trends can be reasonably fitted using bulk 5 ± 1 wt % S and 0.65 ± 0.05 wt % P. Our fractional crystallization models contrast with a prior model based on HSEs (*24*), in which the Re, Os, Ir, Ru, Pt, and Pd concentrations could not be fitted using a single S content. The HSE-based model (*24*) did not exclude any IIF irons in the modeling. They assumed that the core had 11 to 15 wt % S and CI chondritic bulk compositions and that, consequently, Corowa, Dorofeevka, Del Rio, and Repeev Khutor were products of nonequilibrium mixing of primitive solid and evolved liquid, reflecting a more complex crystallization process. In our models, Dorofeevka, Del Rio, and Repeev Khutor are equilibrium solids, and the deviation of Corowa from the IIF solid-liquid field cannot be explained solely by nonequilibrium mixing. Our modeling shows that the four IIF irons represent ≤61% crystallization of the core. The core has low amounts of trapped melt (<10%).

### Group IVB

**Fig. 4.**
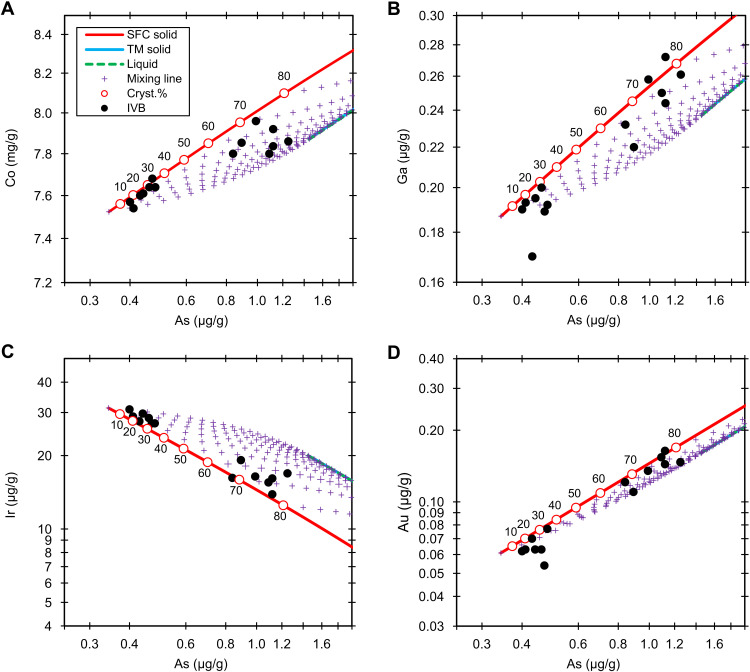
Group IVB. Fractional crystallization model (0.01 wt % S and 0.47 wt % P) of the Co (**A**), Ga (**B**), Ir (**C**), and Au (**D**) versus As in group IVB. The black dots are the NAA data. The red lines, blue lines, and green dashed lines denote the solid derived from simple fractional crystallization (SFC solid), solid from trapped melt (TM solid), and liquid (liquid), respectively. The purple crosses are the mixing lines (mixing line) between fractional crystallization and trapped melt solids at an increment of 5%. The labeled circles on the red lines represent the crystallization sequence (cryst.%). The liquid and trapped melt tracks overlap due to the S-free model conditions.

Group IVB can be best fitted using ~0 wt % S and 0.47 wt % P. The compositional data of Ru, Os, Pt, Mo, Pd, and Rh are partly or entirely from the literature (*27*). The element versus As trends can generally be fitted using 0.5 ± 0.5 wt % S and 0.45 ± 0.02 wt % P. This group has been modeled by several studies (*25*–*27*); all pointed to similarly low S contents (~0 to 2 wt % S). Our model shows that the extremely low S content works for almost all element versus As trends of the group, except that Cu, Ge, Rh, and W versus As trends are slightly scattered beyond the trapped melt model envelopes (Fig. 4 and fig. S5). The relatively high analytical uncertainties of Ge and W account for their scatter.

IVB irons represent ≤78% crystallization of the parent melt. A prior simple fractional crystallization model (without the consideration of trapped melt) shows that IVB irons are products of 17 to 86% fractional crystallization (*25*). In our models, the IVB core generally has low amounts (<15%) of trapped melt; one sample, Ternera, likely has up to 35% trapped melt.

### South Byron Trio

**Fig. 5.**
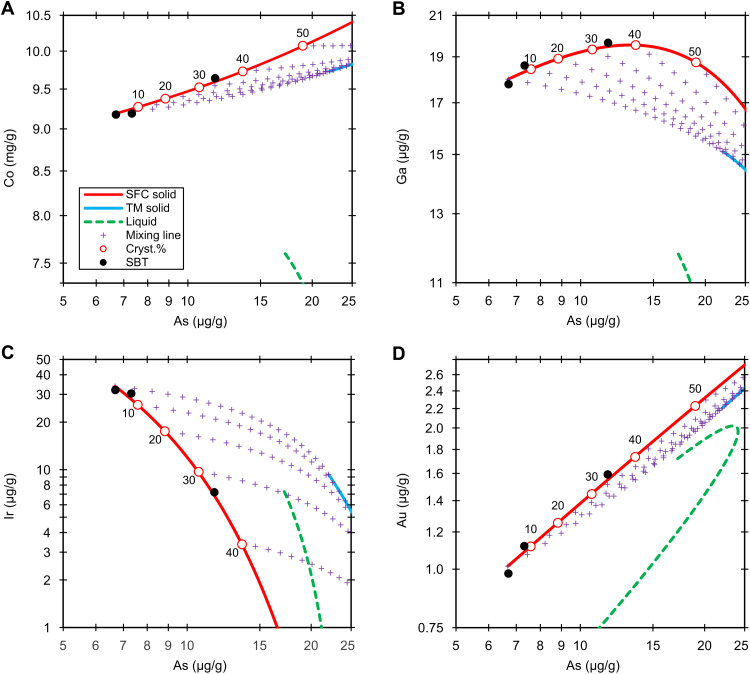
SBT. Fractional crystallization model (8 wt % S and 1.5 wt % P) of the Co (**A**), Ga (**B**), Ir (**C**), and Au (**D**) versus As in the SBT. The black dots are the NAA data. The red lines, blue lines, and green dashed lines denote the solid derived from simple fractional crystallization (SFC solid), solid from trapped melt (TM solid), and liquid (liquid), respectively. The purple crosses are the mixing lines (mixing line) between fractional crystallization and trapped melt solids at an increment of 5%. The labeled circles on the red lines represent the crystallization sequence (cryst.%).

The trio consists of South Byron, Babb’s Mill (Troost’s Iron), and Inland Forts 83500. It can be best fitted using bulk 8 wt % S and 1.5 wt % P (Fig. 5 and fig. S6). The compositional data for Ge, Mo, Ru, Rh, Pd, W, Os, and Pt are partly or all from the literature (table S1) (*43*). The S-bracketing modeling shows that the element versus As trends of the SBT can be fitted with bulk 8 ± 2 wt % S and 1.5 ± 0.3 wt % P. Our results are in line with the bulk S (7 wt %) and P (1 wt %) derived from the modeling of Re, Os, Ir, Ru, Pt, and Pd (*28*). Our model works well for almost all elements with nearly no consideration of trapped melt, while W and Rh have some variation in Babb’s Mill (Troost’s Iron) and South Byron (fig. S6). The SBT represents ≤37% crystallization of the parent melt. This result is close to the number of 1 to 42% crystallization products reported by the prior HSE-based model (*28*). The SBT core has the lowest amounts (<5%) of trapped melt among all CC-iron cores, but, because of the small number of samples, the modeling results must be considered uncertain.

## DISCUSSION

### HSE budgets

**Fig. 6.**
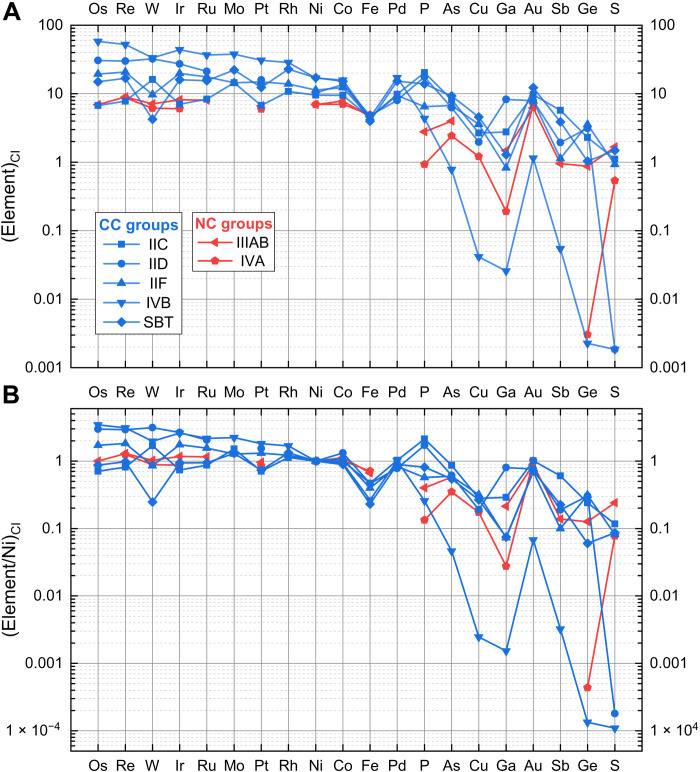
Bulk compositions of the CC-iron meteorite parent bodies. (**A**) Bulk compositions normalized to CI chondrites. (**B**) Bulk compositions normalized to Ni and CI chondrites. The optimal S contents of groups IVB and IID are close to 0, and a value of 0.01 wt % is used here for the two groups to show the approximate position of S. Composition of CI chondrites from the literature (*69*). Elements are arranged in decreasing order of their *T*_50_ (*44*).

Figure 6 plots our results for the bulk compositions, and element/Ni ratios of all CC-iron cores normalized to CI chondrites, arranged in order of their 50% condensation temperatures (*T*_50_) (*44*). The bulk Os and Re concentrations in IIC, SBT, IIF, IID, and IVB cores are ~7×, ~15×, ~20×, ~30×, and ~ 55 × CI, respectively (Fig. 6A). The elevated bulk concentrations of HSEs in a core are due either to the redox state of the parent body (*28*) (affecting the core/mantle ratio) and/or the admixture of different abundances of high-temperature refractory metals from the solar nebula (*25*, *43*). The latter scenario was first proposed to explain the extremely high HSE abundances in group IVB (*25*). In carbonaceous chondrites, HSEs are greatly enriched in CAIs compared with other silicate-rich components (i.e., chondrules, matrix, and isolated mafic silicate grains) (*45*), and submicrometer- to micrometer-scale refractory metal nuggets (RMNs) are the primary host for HSEs (*46*). RMNs inherited from melted CAIs have been proposed as the main source of HSEs in some CC-iron cores (*47*).

An important sign of the influence of RMNs in the parent melt of an iron meteorite core (e.g., group IVB) is that its Ni- and CI-normalized abundances of HSEs versus *T*_50_ would form a decreasing slope, because these elements condensed at different temperatures as the nebular gas cooled from high temperatures (*25*). In contrast to the decreasing slope of group IVB on the (HSE/Ni)_CI_ versus *T*_50_ diagram, the slopes of group IIC and the SBT are nearly flat (Fig. 6B). If the precursors of these two cores were CI chondritic, then flat (HSE/Ni)_CI_ patterns would be expected because HSEs would have fully condensed at the lower temperatures where Fe and Ni dominate the core (*25*). The chondritic HSE abundances and patterns of group IIC and the SBT indicate that their parent melts had minimal admixtures of RMNs. The enrichment of bulk HSEs in the IIC and SBT cores compared with those in CI chondrites is due mainly to lower reduced Fe in the core, indicating a high oxidation state of their parent bodies (*23*).

The (HSE/Ni)_CI_ values in the IID and IVB parent cores are both ~3 × CI (Fig. 6B). These high Ni- and CI-normalized HSE abundances do not seem to have been caused solely by a high oxidation state in their parent asteroids. For example, the IVB core has a Ni concentration (178 mg/g) similar to that of the SBT core (180 mg/g), but the IVB core has >3× higher (HSE/Ni)_CI_ values than the SBT core. The high (HSE/Ni)_CI_ values of group IID are accompanied by a slightly sloping pattern in Fig. 6B; the pattern resembles that of group IVB (*25*). We, therefore, interpret the high (HSE/Ni)_CI_ values in the IID core as a result of the admixture of RMNs. The HSE enrichment and sloping pattern are also observed in CK and CV chondrites (*48*), the most CAI-rich chondrite groups with ~4 volume percent (volume %) and ~ 3 volume % CAIs (the main host of RMNs), respectively (*49*).

**Fig. 7.**
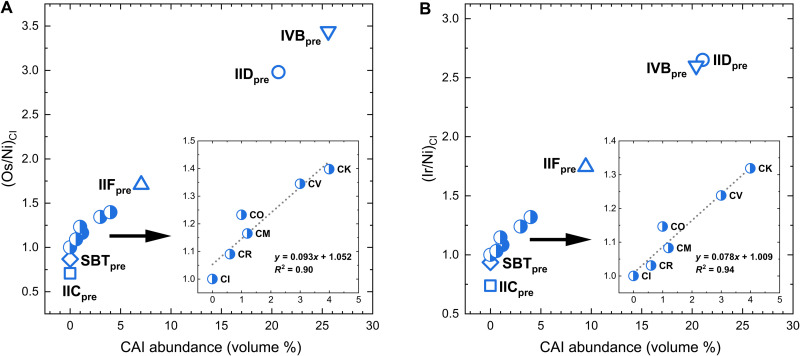
(Os/Ni)_CI_ and (Ir/Ni)_CI_ values versus CAI abundances in carbonaceous chondrites and estimated CAI abundances for CC-iron precursors. The (Os/Ni)_CI_ (**A**) and (Ir/Ni)_CI_ (**B**) values for carbonaceous chondrites show a linear relationship (*R*^2^ ≥ 0.90) with CAI abundances. The carbonaceous-chondrite-like precursors of CC-iron parent bodies are denoted by the group name with a subscript “pre,” and their positions on the diagrams are estimated from our model-derived Os/Ni and Ir/Ni abundances and the linear regression equations for carbonaceous chondrites. CAI abundances of the SBT and IIC precursors are assumed to be CI chondritic. Nickel, Os, and Ir concentrations (*48*) and CAI abundances (*47*) of chondrites are from the literature.

The Ni-normalized HSE abundances of NC groups IIIAB and IVA are chondritic and significantly lower than those of the CC-iron groups IID, IIF, and IVB; however, they are similar to those of group IIC and the SBT. Therefore, the difference in HSE abundance between the CC- and NC-iron cores can be ascribed only partly to the different oxidation states of the two suites (*23*); the extraordinarily high HSE abundances in some CC-iron cores are likely due mainly to the incorporation of RMNs into their parent metallic melts. CAIs are a primary factor that influences the HSE contents in chondrites. Carbonaceous chondrites have higher CI-normalized Os, Ir, and Ru abundances than ordinary chondrites (*48*), consistent with higher CAI abundances in most carbonaceous chondrites than in ordinary chondrites (*49*). The CAI abundances among carbonaceous chondrites vary widely (CI, 0 volume %; CM, 1.2 volume %; CO, 1.0 volume %; CV, 3.0 volume %; CK, 4 volume %; CR, 0.6 volume %) (*49*). CAI abundances show a highly linear relationship [*R*^2^ (coefficient of determination) ≥ 0.90] with the HSE abundances in carbonaceous chondrites (Fig. 7). The variability in CAI abundance also plausibly occurred in the carbonaceous-chondrite-like precursors of CC-iron parent bodies and could account for the large variation in (HSE/Ni)_CI_ values of CC-iron cores.

The high (HSE/Ni)_CI_ values of IVB and IID cores cannot be explained by the CAI abundances in the set of carbonaceous chondrites currently in meteorite collections. It is plausible that the IVB and IID parent bodies were derived from carbonaceous-chondrite-like precursors with CAI abundances higher than those of CV and CK chondrites. Using the linear relationship between HSE abundance and CAI abundance in carbonaceous chondrites (Fig. 7), we estimate ~20 volume % CAIs for the precursors of the IVB and IID parent bodies; this is equivalent to ~26 wt % [assuming a density of 3.1 g/cm^3^ for CAIs (*50*) and 2.2 g/cm^3^ for their CI chondrite–like host matrices (*51*)]. Our estimate is consistent with the high CAI abundances (~30 ± 10 volume %) inferred from spectroscopic observations of several asteroids (*52*).

Both CC and NC meteorites show large variations in *s*-process Mo isotopes, and CC meteorites show uniform *r*-process excess over NC meteorites (*1*, *5*). These variations in Mo nucleosynthetic components cause CC and NC meteorites to form nearly parallel *s*-process mixing lines with a resolvable offset on the ε^95^Mo versus ε^94^Mo diagram (*1*, *5*). Assuming the relative enrichment of HSEs in some CC-iron groups resulted from their parent bodies having incorporated greater proportions of *r*-process–enriched CAIs, one would expect that IVB and IID irons might plot above the CC *s*-process mixing line on the ε^95^Mo versus ε^94^Mo diagram (*53*). Specifically, if the *r*-process–enriched unfractionated CAIs (*54*) are the dominant type of CAIs in the IVB precursor, then these CAIs (mean ε^95^Mo = 1.9 ± 0.3) (*54*) would increase the ε^95^Mo value of IVB irons (1.16 ± 0.05) (*55*) by ~0.4 using a CAI abundance of 26 wt % in the precursor and a relative core mass of 10% in the parent asteroid. However, IVB and IID irons do not plot above the CC line (*5*). One possible explanation is that the CAIs incorporated into the IVB and IID cores were not *r*-process enriched (*54*). Alternatively, incorporation of some *r*-process–deficient RMNs (*56*) into the cores could partly offset the effect of *r*-process–enriched CAIs.

### Sulfur and P abundances

**Fig. 8.**
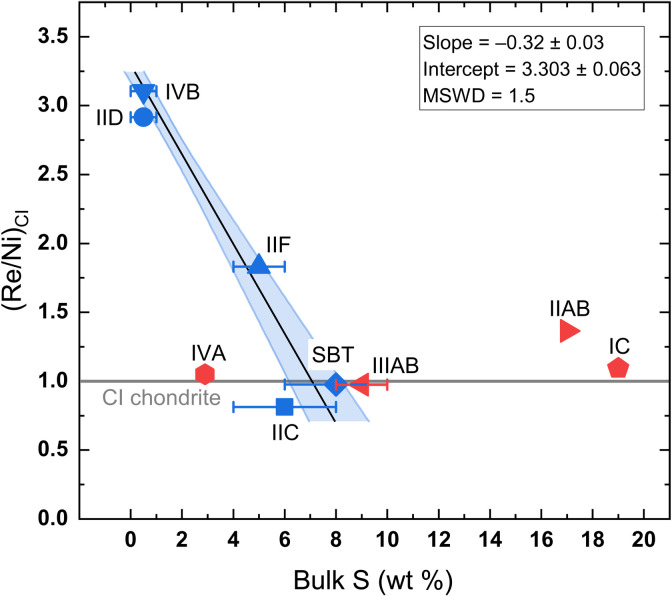
Bulk S concentrations plotted against Ni- and CI-normalized Re concentrations. Rhenium is used as a representative element of HSEs. Data of NC-iron groups IC (*23*, *29*), IIAB (*23*, *26*), IIIAB (*32*), and IVA (*35*) are in red symbols. The CC-iron groups are blue symbols. The solid line is the maximum-likelihood fit with 1σ error envelope for the CC-iron groups. Composition of CI chondrites from the literature (*69*). MSWD, mean square weighted deviation.

Bulk S contents (0 to 8 wt %) of the CC-iron cores are lower than those of several NC-iron cores: IC (19 wt %) (*29*), IIIAB (9 ± 1 wt %) (*32*), and IIAB (17 wt %) (*26*); whereas the NC-iron IVA core (2.9 wt % S) (*35*) is in the range of CC-iron cores. The S concentrations of the CC-iron cores are anticorrelated with the relative HSE abundances, but the anticorrelation is not observed in NC-iron cores (Fig. 8). The general difference in bulk S content between the CC- and NC-iron cores may be related to the variability in S content among the precursors of iron meteorite parent bodies in the CC and NC reservoirs (*28*). The bulk S content affects the temperature at which the metallic composition is fully molten and, hence, the timing of differentiation of an asteroid. This is consistent with the anticorrelation of bulk S contents and ε^182^W values of iron meteorite cores (fig. S7) (*28*, *57*). Our results further support the idea that S contents may have exerted an important influence on the differentiation temperature of iron meteorite parent bodies (*57*).

The P contents of CC-iron cores are noticeably higher than those of the NC-iron IIIAB and IVA cores (*23*). The mean bulk P concentrations of CC-iron cores is 1.3 wt % (Table 1), whereas averaged NC-iron cores contain 0.3 wt % P (determined by modeling the HSEs of four NC-iron cores). This P abundance difference is opposite to the S abundance difference in the CC- and NC-iron cores.

### Fractionation of volatile and moderately volatile siderophiles

The CI-normalized abundances of volatile and moderately volatile siderophiles (P, As, Cu, Ga, Au, Sb, Ge, and S) in iron meteorites generally decrease along with the *T*_50_ of the elements, although the patterns are scattered (Fig. 6A). Nickel-normalized volatile and moderately volatile siderophiles in the CC-iron cores are depleted relative to CI chondrites (Fig. 6B). The depletion of volatile siderophile elements in the CC-iron cores may have been inherited from their carbonaceous-chondrite-like precursors, and differentiation and crystallization processes may have further shaped the volatile inventory (*58*).

Devolatilization of iron meteorite cores during crystallization could also modify volatile abundances (*59*). Despite having a similar sloping pattern, group IVB has significantly lower (volatile/Ni)_CI_ values than any other iron meteorite group. The (volatile/Ni)_CI_ discrepancy between IVB and other groups increases as a function of volatility (Fig. 6B). These low abundances of volatile siderophiles in group IVB are attributable to high-temperature processes in the solar nebula that fractionated HSEs (*25*) or to crystallization processes including catastrophic disruption during the crystallization of the core (*60*). If the low volatile abundances of group IVB resulted from nebular processes, then other HSE-rich groups would display similar extreme depletion of volatiles. However, group IID, with similar (HSE/Ni)_CI_ values to those of group IVB, has several orders of magnitude higher (volatile/Ni)_CI_ values (Fig. 6B). Therefore, our results imply that the great depletion in volatiles of the IVB core is more likely to have been caused by devolatilization of the parent body after accretion.

Early loss of a S-rich melt could lower the abundances of S and some other siderophile elements within an iron meteorite core. Group IID has the highest bulk P/S ratio and the highest Ga and Ge concentrations among all magmatic iron groups (*22*). The bulk composition of the IID parent melt is also consistent with a chondritic pattern (Fig. 6), suggesting that this melt did not form from an evolved, late-stage P-rich liquid, as has been suggested for the P-rich IIG irons (*61*, *62*). A possible explanation for the high bulk P/S ratio of the IID parent body is that it formed metastable liquid layers due to episodic melting during differentiation, during which a low-temperature S-rich melt was removed from the parent melt when the metallic melt reached the Fe-FeS eutectic at the relatively low temperature of ~1220 K (*18*, *22*). In that case, most siderophile elements would have remained in the solid, while chalcophile elements would have partitioned into the S-rich melt. Copper (chalcophile) and Ga (siderophile) have similar *T*_50_ of 1034 and 1010 K, respectively (*44*). Within a single iron meteorite group, the extraction of a S-rich melt would lower the Cu/Ga ratio of the group. The Cu_mean_/Ga_mean_ ratio of IID irons is more than two times lower than those of other CC-iron groups (table S1). We hence attribute the high P/S ratio of group IID to the early extraction of a S-rich melt; such a process caused S and Cu depletions in the core.

The (volatile/Ni)_CI_ patterns of the CC-iron groups are generally similar to the two NC-iron groups, whose bulk compositions have been modeled by previous studies for similar elements (Fig. 6B). The depletion and fractionation patterns of other volatile and moderately volatile siderophiles do not clearly distinguish CC- and NC-iron cores.

### Crystallization processes

Our models show that all CC-iron groups represent incomplete sampling of their cores, as summarized in Table 1. Because several CC-iron groups (or grouplets) have only a few members, incomplete sampling of the crystallization sequence is expected, and the CC-iron groups with the most current INAA-analyzed members (IID, 23 members; IVB, 14 members) should represent the most complete sampling of the crystallization sequences. However, in all cases, the latest crystallization products are missing. The late crystallization products, especially for the IIC and SBT cores with their high initial S contents, are inferred to be S rich. These S-rich materials have been suggested to be rare in our collections due to attrition in interplanetary space, high ablation rates during atmospheric passage, and more rapid terrestrial weathering (*18*). Irons that formed close to or at the Fe-FeS eutectic may have been prone to fragmentation or too small to make it into our museum collections (*22*). The fragility of FeS-rich irons may explain our observation that only the early crystallization sequences were sampled by the high S IIC (≤30%) and SBT (≤37%), although the paucity of members in the group/grouplet is also a limitation. For the very low S IID (crystallization sequence of ≤84%) and IVB (≤78%) cores, our modeling shows that even-more-evolved finds would have formed from liquids with very low S (≤1 wt %). It is possible that future finds of these two groups could represent later-crystallized core samples. Similar to the case of the CC-iron groups, the NC-iron groups IC, IIAB, and IIIAB are incomplete samplings of their cores, respectively, representing the first ~13% (*29*), 49% (*30*), and ~60% (*32*) crystallization products of their parent melts. The incomplete sampling of the NC-iron groups (especially the largest groups—IIAB and IIIAB) supports the idea that a considerable fraction of the high bulk S cores is not sampled by our current collections.

Our models also show that the current collections of the CC-iron groups are derived from either equilibrium solids or equilibrium mixing of solids and liquids instead of from nonequilibrium processes. The interelement trends of all CC-iron groups can be explained by fractional crystallization models with minor equilibrium mixing, with small amounts of trapped melt (<15%) needed for fitting the interelement trends. This indicates that the CC-iron cores crystallized from well-mixed metallic melts and, hence, did not form substantial dendrites or impact-induced cracks (*31*) that hindered global convection.

In contrast, trapped melt models have been applied to several NC-iron groups, such as IIAB (*30*), IIIAB (*31*, *32*), and IVA (*33*–*35*); all these groups show significantly high amounts (up to 100%) of trapped melt. The formations of trapped melt and dendrites in the NC-iron cores would have affected their crystallization processes. The distinct difference in amounts of trapped melt between the NC-iron and CC-iron groups, as determined from their geochemical properties, suggests that the iron meteorite cores from the two reservoirs may have displayed some geophysical differences, such as core sizes and mechanical properties, during crystallization.

### Early HSE heterogeneity in the CC reservoir

Our bulk HSE data for CC-iron cores suggest that variations in the enrichment of HSEs in the CC reservoir were present. If the HSE enrichment observed in some CC-iron cores is due to enrichment of RMNs, then this has implications for the timing of early protoplanetary disk evolution. The RMNs are high-temperature condensates that formed very early in solar system history, most likely close to the Sun. The RMNs, together with their host CAIs, were transported outward during the early viscous expansion of the disk and later drifted back toward the Sun (*7*). To account for the relatively high abundances of CAIs in some carbonaceous chondrites (e.g., CV and CK), it has been suggested that the inward drift of CAIs was blocked by a pressure bump formed by the early accretion of Jupiter (*63*). In this pressure bump, inward drifting CAIs could accumulate for several million years, resulting in the enrichment of CAIs observed in some carbonaceous chondrites (*63*). Because the CC-iron parent bodies accreted within ~1 Ma after CAI formation (*5*, *16*), our study suggests the outward RMN enrichment in the protoplanetary disk occurred even earlier.

**Fig. 9.**
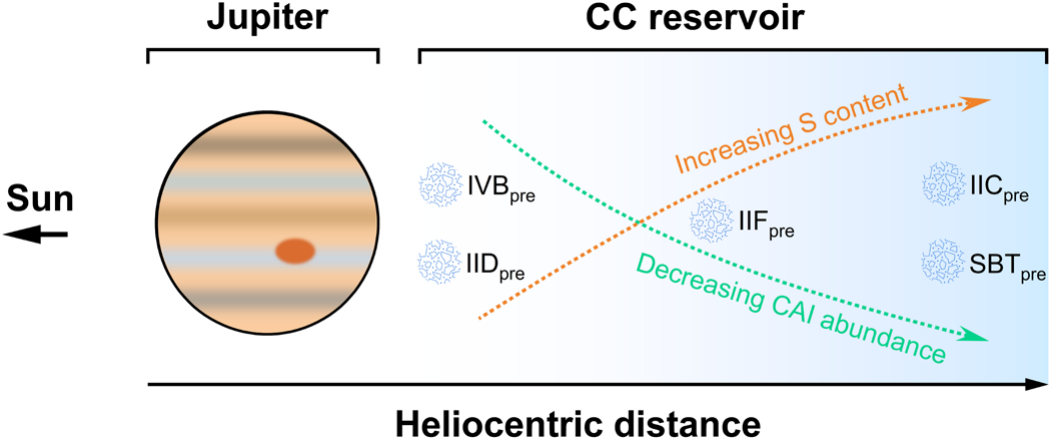
Relative spatial distribution of precursors of CC-iron parent bodies at <1 Ma after CAI formation. The carbonaceous-chondrite-like precursors of CC-iron parent bodies are denoted by the group name with a subscript pre. The positions of the precursors denote their relative heliocentric distance in the disk suggested by our study results. The results of our study suggest that the S contents and CAI abundances of the precursors increase and decrease, respectively, as the heliocentric distance increases.

The abundance of CAIs in the early CC reservoir decreases as the heliocentric distance increases (*63*). Specifically, CV and CK chondrites are predicted to have formed in the pressure bump beyond Jupiter at ~3.6 au (astronomical unit), and CR and CO chondrites formed in the same pressure bump but farther from Jupiter, while CI chondrites originated farther out in the disk (*63*). Taking into account this evolution model of the solar protoplanetary disk and the HSE abundances of CC-iron cores estimated by our fractional crystallization models, we suggest that the HSE-enriched IID and IVB asteroids formed close to Jupiter in the pressure bump, and the HSE chondritic IIC and SBT asteroids formed farther from Jupiter (Fig. 9). The IIF asteroid likely accreted in a region between those of the HSE-rich and HSE chondritic asteroids (Fig. 9). [However, the evolution model predicts a maximum CAI abundance of 5.9 wt % in the pressure bump (*63*), lower than our estimate (~26 wt %) for the IVB and IID precursors.]

The S contents in the CC reservoir in the protoplanetary disk may have also been tied to formation location in the disk, with increasing S contents at larger heliocentric distances. Such an increasing S distribution pattern is suggested in carbonaceous chondrites (CO, 2.0 wt %; CV, 2.2 wt % S; CM, 3.3 wt % S; CI, 5.9 wt % S) (*48*). Our estimates for the S contents of CC iron meteorite parent bodies also follow this increasing S distribution pattern. We suggest the low-S IID and IVB cores formed within asteroids close to Jupiter, followed by the IIF parent body with a core containing ~5 wt % S at an intermediate distance, and the parent asteroids of the IIC (~6 wt % S) and SBT (~8 wt % S) cores farthest from Jupiter (Fig. 9). Thus, the negative linear relationship between HSE abundance and S content of CC-iron cores in Fig. 8 may be due to spatially correlated compositional variations that were established within the CC reservoir very early in the solar system, within ~1 Ma after CAI formation. Fine-grained silicate matrix material in chondrites contains most of the volatile components in the whole rock (*64*). Aerodynamic processes may have caused CC planetesimals accreting near Jupiter to acquire higher modal ratios of coarse particles (e.g., CAIs) to fine grains (matrix). This would have resulted in these bodies containing higher concentrations of HSEs and lower concentrations of S.

### Summary

The bulk compositions and crystallization processes of CC-type iron meteorite cores provide insights into the evolution of metallic melts and asteroidal cores in the first few million years of solar system history. The bulk composition and crystallization processes were reconstructed from fractional crystallization modeling of the interelement trends of CC-type iron meteorite groups.

The modeling of the CC-iron groups, along with currently available modeling of the NC-iron groups, shows that the dichotomy in isotopic compositions of the two reservoirs may also pertain to redox conditions, bulk chemical compositions, crystallization processes, and compositional evolution. The CC-iron cores crystallized in more oxidized environments than the NC-iron cores (*23*, *47*). The CC-iron cores have lower S, higher P, higher Ni, and, in some cases, higher HSEs in their parent melts than the NC-iron cores. The CC-type cores seem to have crystallized from well-mixed melts and did not develop significant amounts of trapped melt or dendrites that would have impeded global convection; this contrasts with the NC-iron groups IIAB, IIIAB, and IVA that underwent more complicated crystallization processes (e.g., formation of large amounts of trapped melt and/or a network of dendrites).

The fractional crystallization models for four CC-iron groups and one grouplet show that they sample a small-to-high fraction of the crystallization sequence of their cores; the latest crystallization products are missing in current meteorite collections. In the accretion processes of the CC iron meteorite parent bodies, considerable amounts of volatile and moderately volatile elements were lost due to their lower condensation temperatures than those of Ni and Fe. Devolatilization during the crystallization of an asteroid (specifically group IVB) could have further depleted the volatile and moderately volatile elements in the core.

Many of the CC-iron cores have high bulk HSE abundances relative to CI chondrites, partly caused by the high oxidation state of the CC-iron cores. We suggest that the exceptionally high CI chondrite– and Ni-normalized HSE abundances of groups IID and IVB are due to the incorporation of RMNs into their metallic melts, inherited from melted CAIs in their carbonaceous-chondrite-like precursors. The early enrichment of CAIs (~1 Ma after CAI formation) in the CC reservoirs indicated by CC-iron cores may require that a substantial fraction of Jupiter accreted by this time. By analogy to a similar model accounting for the variation in CAI abundance in carbonaceous chondrites (*63*), the HSE-rich IVB and IID parent asteroids likely accreted in the pressure bump close to Jupiter. The IIF asteroid accreted a little farther out in the disk. The HSE chondritic IIC and SBT cores developed within asteroids that accreted even farther out in the disk. The precursors of the IVB and IID parent bodies may be more enriched in CAIs than those of the current carbonaceous-chondrite classes.

## MATERIALS AND METHODS

The elemental concentrations of Cr, Co, Ni, Cu, Ga, As, Ru, Sb, Os, Re, Ir, Pt, and Au were analyzed by INAA; most of the Ge and Sb data used in the mean calculations were obtained by radiochemical NAA (RNAA). Some Ni replicates determined by atomic absorption spectrometry are from the literature (*41*, *65*–*67*). The INAA methods are described in the literature (*30*, *68*). Data for Fe were used for internal normalization in the INAA procedures. To improve the analytical precision and reduce analytical uncertainties, irons were analyzed at least twice, except for a few meteorites with a total mass insufficient to allow replicate analyses. All irons were sawed into 3.0-mm-thick, 550-mg rectangular specimens. Each specimen has minimal contents of nonmetal impurities or inclusions. Specimens were irradiated for 4 hours in the reactor at the University of California, Irvine. After irradiation, irons were leached with dilute H_2_SO_4_, HCl, and HNO_3_ to remove superficial contamination. Specimens were counted four times on a hyperpure planar Ge detector for 6, 15, 80, and 600 hours, respectively, over a period of 4 weeks. Sample-specific geometric corrections (0.95 to 1.05) to the Ni and Co values were made to be consistent with the third and fourth counts, in which Fe + Ni was corrected to be 990 mg/g. The INAA data were processed using in-house software. The pretreatment of specimens, counting, and data processing were performed at the University of California, Los Angeles (UCLA).

In analyses conducted before 1986, the Filomena specimen of North Chile (IIAB) was not used as a standard; instead, aliquots of standardized solutions were used. In the past two decades, Filomena, Coahuila (IIAB), and NBS steel NBS809B were used as standards. Some of the older data were recalculated by restandardizing them with newer analyses; in each case, the recalculations make less than a 5% difference. Concentrations of Ge were previously determined by RNAA because Ge concentrations were below INAA detection limits. Therefore, the Ge RNAA data shown in this study are mainly from the literature (*65*–*67*). Gallium data are calculated both from our INAA data and from RNAA data from the literature (*65*–*67*). In the text, NAA refers to either INAA or RNAA.

The relative 95% confidence limits on the mean values in tables S1 and S2 are as follows: 1.5 to 3% for Co, Ni, Ga, Ir (concentrations, >0.1 μg/g), and Au; 4 to 6% for As, Ge (by RNAA), and Sb; and 7 to 10% for W (values, >0.3 μg/g), Re (>50 ng/g), Ru (>4 μg/g), and Pt (>2 μg/g). Chromium typically occurs in minor phases such as chromite and daubréelite; this sampling bias is responsible for the high relative confidence limits (>10%) on the means of Cr. In addition, Fe produces interference in the determination of Cr due to the ^54^Fe(n,α)^51^Cr fast neutron reaction. The degree of interference is about 6 μg of Cr per gram of Fe (*33*).

The NAA data were collected over the past four plus decades; starting in 1986, the data quality improved significantly. Replicates analyzed after 1986 were given 1.5 to 2× weight in the mean calculation. The means of all iron meteorites are shown in table S1.

In the fractional crystallization modeling, we used NAA data and extended it to more elements with data from isotope dilution (ID)– or LA-ICP-MS. In the INAA analyses performed at UCLA, we irradiated one ~500-mg block of each iron meteorite to acquire one replicate, and the mean composition of each iron meteorite was calculated from two to four replicates (blocks sawed from different parts of the meteorite). All replicates of one iron meteorite were matched to examine the compositional homogeneity of the meteorite. Our INAA data should represent bulk measurements of the metal in iron meteorites. When NAA data of some elements were absent, we used ID-ICP-MS data from the literature (*21*, *23*, *27*, *28*), except that we prioritized Pt and Ru data by ID-ICP-MS in the modeling due to their higher analytical precision compared with that of INAA. LA-ICP-MS analyses for irons are commonly based on combining data from hundreds of micrometer-scale spot measurements. Even with the analysis of multiple “tracks,” this technique relies on averaging data from an area smaller than the amount of material used for NAA, which can increase the potential influence of sample heterogeneity in bulk measurements. We, therefore, used only LA-ICP-MS data from the literature when NAA or ID-ICP-MS data were not available. The fractional crystallization modeling methods are detailed in the Supplementary Materials.

## Supplementary Materials

### This PDF file includes:

Supplementary Text Figs. S1 to S7 Tables S1 to S4 References
