# Supplementary material for: Compositions of carbonaceous-type asteroidal cores in the early solar system
Source: Sci Adv. 2022 Sep 16;8(37):eabo5781. doi: 10.1126/sciadv.abo5781 (PMC9481128; doi:10.1126/sciadv.abo5781)
Supplement: Supplementary file 1 — Supplementary Text Figs. S1 to S7 Tables S1 to S4 References [file sciadv.abo5781_sm.pdf]

Science Advances
AAAS

Supplementary Materials for

Compositions of carbonaceous-type asteroidal cores in the early solar system

Bidong Zhang *et al*.

Corresponding author: Bidong Zhang, bdzhang@ucla.edu

Sci. Adv. **8**, eabo5781 (2022)
DOI: 10.1126/sciadv.abo5781

This PDF file includes:

Supplementary Text
Figs. S1 to S7
Tables S1 to S4
References

Supplementary Text

Modeling methods

The fractional-crystallization modeling methods are described in the literature (*26, 32*). We provide a short summary below. In brief, the modeling uses batch crystallization in small steps to simulate fractional crystallization of metallic melts. This is because, in metallic melts, the partition coefficients of trace elements change as the liquid composition (especially S and P concentration) changes during crystallization. The remaining liquid in each step is used as the starting liquid of the next step.

Equilibrium batch crystallization is a simple mass balance between the phase fields:

$$\frac{C_L}{C_i} = \frac{1}{(1 - f + f \times D_E)} \quad (1)$$

In Eq. 1, C_i, C_L, f, and D_E represent the bulk composition of the liquid, the bulk composition of the remaining liquid, the crystallization step, and the partition coefficient of an element, respectively. The models in this study used constant 0.001 for each mass step. The concentration of an element in the solid (C_s) derived from each mass step is calculated using the bulk composition of the remaining liquid and the partition coefficient of the element in that step:

$$C_s = D_E \times C_L \quad (2)$$

The partition coefficient of an element is strongly influenced by the S and P contents of the liquid and varies at each small step. D_E is parameterized using Eq. 3 (*26*).

$$D_E = D_0 \times (Fe\ domains)^{\beta} \quad (3)$$

D_0 is the partition coefficient of an element in the S- and P-free system. β is a constant specific to an element related to S and P in the liquid. *Fe domains* represent the fraction of free Fe atoms available in the liquid (*37*). *Fe domains* in the Fe-Ni-S-P system were calculated by Eq. 4, and β_{S+P} of an element in the Fe-Ni-S-P system was calculated using Eq. 5 (*20*).

$$Fe\ domains = \frac{1 - 2X_S - 4X_P}{1 - X_S - 3X_P} \quad (4)$$

$$\beta_{S+P} = \left[\frac{2X_S}{(2X_S + 4X_P)}\right]\beta_S + \left[\frac{4X_P}{(2X_S + 4X_P)}\right]\beta_P \quad (5)$$

X_S and X_P are the molar fractions of S and P in the liquid, respectively. β_S and β_P are the beta values for each element in the Fe-S and Fe-P systems, respectively.

The scattered interelement trends of group IIIAB can be caused by the equilibrium mixing of solid and liquid (trapped melt) (*31*), which is called the trapped-melt model. A recently revised version of the trapped-melt model considers the formation of troilite in the trapped melt (*32*). The relationship between the trapped melt ($C_{Trapped\ melt}$) and the solid ($C_{Trapped\ melt\ solid}$) that crystallized from the trapped melt can be expressed using Eq. 6:

$$C_{Trapped\ melt\ solid} = \frac{C_{Trapped\ melt}}{1-x} \quad (6)$$

where x denotes the mass fraction of the trapped melt that solidifies to form troilite. In this study, we consider the formation of all groups and evaluate the fraction of trapped melt for each group.

We used the composition data of Cr, Co, Ni, Cu, Ga, Ge, As, Sb, Ru, Re, Os, W, Ir, and Au determined by NAA. Some Ru and Os data and all Rh, Pd, and Mo data are from ICP-MS data in the literature. Phosphorus concentrations are from modal analyses (*70*). Details of the data sources are shown in table 1. The models in this study are based on element vs. As trends. The use of As and Au as abscissa in the fractional-crystallization models was first used in group IIIAB (*31*). Arsenic and Au have almost the same geochemical behavior during fractional crystallization in metallic melts (*37*). These two elements have lower partition coefficients so that they have a larger range of concentrations. For an iron group, the range of Au and As concentrations is larger than their INAA analytical uncertainties (1.5 to 3% for Au, 4 to 6% for As) (*22*). As a result, element-Au and element-As diagrams provide better estimates of the position of meteorites in the fractional-crystallization tracks. Despite the almost identical behaviors of As and Au during fractional crystallization, the partition coefficient of Au is relatively poorly understood in low-S melts (*32, 37*). Thus, element vs. As trends were used to evaluate our fractional-crystallization models.

The bulk compositions of the CC-type groups were determined by the trial-and-error method. The first solids obtained by the models are assumed to be the lowest-As irons or solids with similar compositions. We assume the lower boundary of the envelope of an element-As trend overlaps with the SFC solid track. The initial elemental concentrations are adjusted to fit the model tracks with as many element-As trends as possible at the same time. An optimal initial S content is thereby obtained. The adjacent sulfur contents bracketing the optimal S content are tested at increments of 0.5% (groups IVB and IID) or 1% (other groups), enabling us to take the analytical and modeling uncertainties into consideration in the models.

<u>Comparison between our model and the HSE-based model for group IID</u>

A recent study used the same modeling method (based only on HSEs) for group IID and resulted in initial bulk concentration estimates of 10 wt % S and 1 wt P (*23*); this S content is drastically different from our S value of 0.5 ± 0.5 wt % and a prior estimate of 0.7 wt % (*22*). The bulk Re, Ir, and Pt concentrations in the low-S models (this study and (*22*)) are two to three times higher than those in the high-S model (*23*) (table S4). The crystallization sequence in the BSE-based model is ~20%; the corresponding number in our model is ≤84%. A notable difference between our model and their model is the assumption of how much trapped melt there is in the most evolved iron, Wallapai. In the HSE-based model, this iron meteorite is composed of >90% trapped-melt solid on the Re/Os vs. Re diagram. Our model instead follows the literature (*22*) that Wallapai should be on the solid track because it has very low FeS abundances (*70*) and, consequently, low content of trapped melt. If Wallapai had 90% trapped-melt solid and was a product of 20% crystallization of the core, there should be ~30 wt % troilite in Wallapai. On the Ir vs. As diagram (fig. S1B) using 10 wt % S and 1 wt % P, the trapped-melt content of Wallapai is ~70%, corresponding to ~24 wt % troilite in the metal. However, in Wallapai, "*troilite is conspicuous by its absence. On polished sections totaling 1,200 cm^2 the largest troilite crystal*

was 1 mm across, and inspection of the exterior surface of the two main masses also failed to disclose any pits which could be unequivocally attributed to troilite" (*70*). It cannot be excluded that the polished section was a nonrepresentative sample of Wallapai. However, for the large section, such a low troilite content for an evolved iron is more consistent with the low bulk S content in group IID. Another notable inconsistency in using 10 wt % S is that it cannot explain the Ga vs. As trend (fig. S1A); Wallapai would remain outside the trapped-melt-model envelope on the Ir vs. Au diagram (fig. S1C). Due to the similarity of As and Au in fractional-crystallization parametrization, the Ir vs. Au and Ir vs. As diagrams should show similar crystallization sequences and fractions of trapped melt in an optimal fractional-crystallization model (*22, 30, 31*).

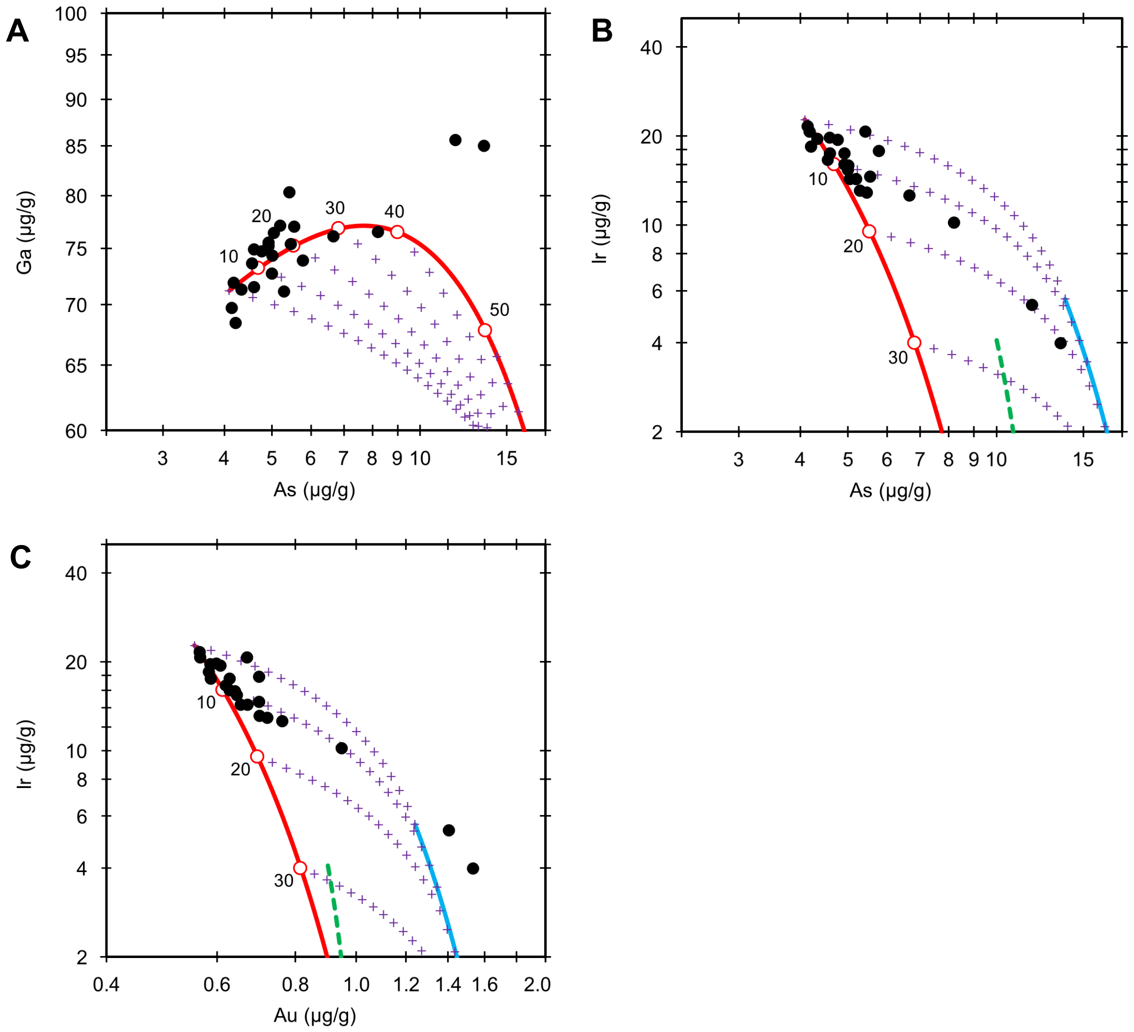

Fig. S1. Ga-As, Ir-As, and Ir-Au trends of group IID using 10 wt % S and 1 wt % P. The bulk Ir concentration is from the HSE-based model with 10 wt % S (*23*), and bulk Ga, As and Au concentrations are adjusted to make the best fit.

Modeling of more elements for groups IIC, IID, IIF, IVB, and the SBT

Figures 1−5 show Co, Ga, Ir, and Au vs. As diagrams, and figs. S2−6 show the rest of the element vs. As diagrams for the CC-iron groups with respective optimal S and P contents. The bulk compositions used in the models are listed in table S3.

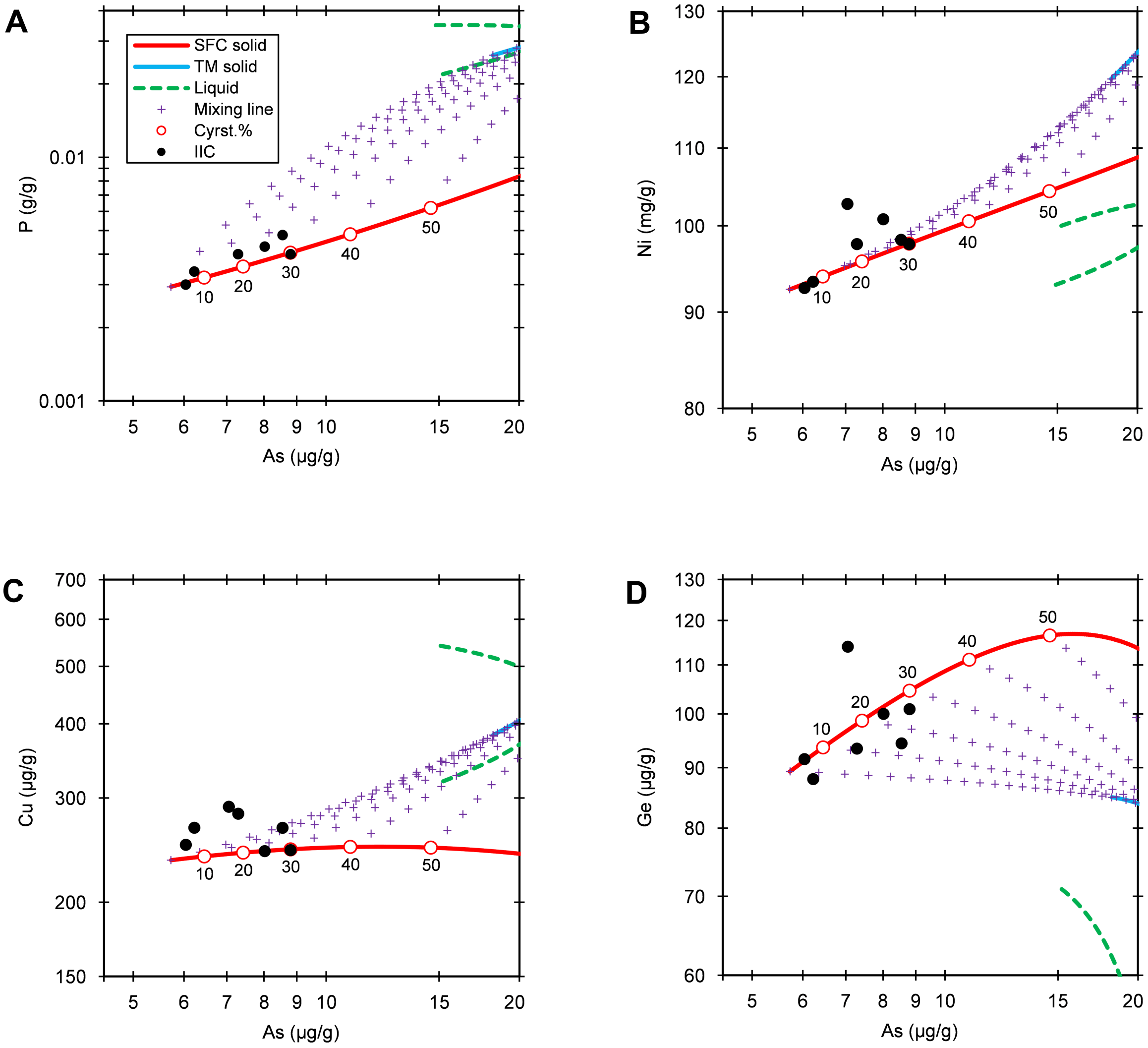

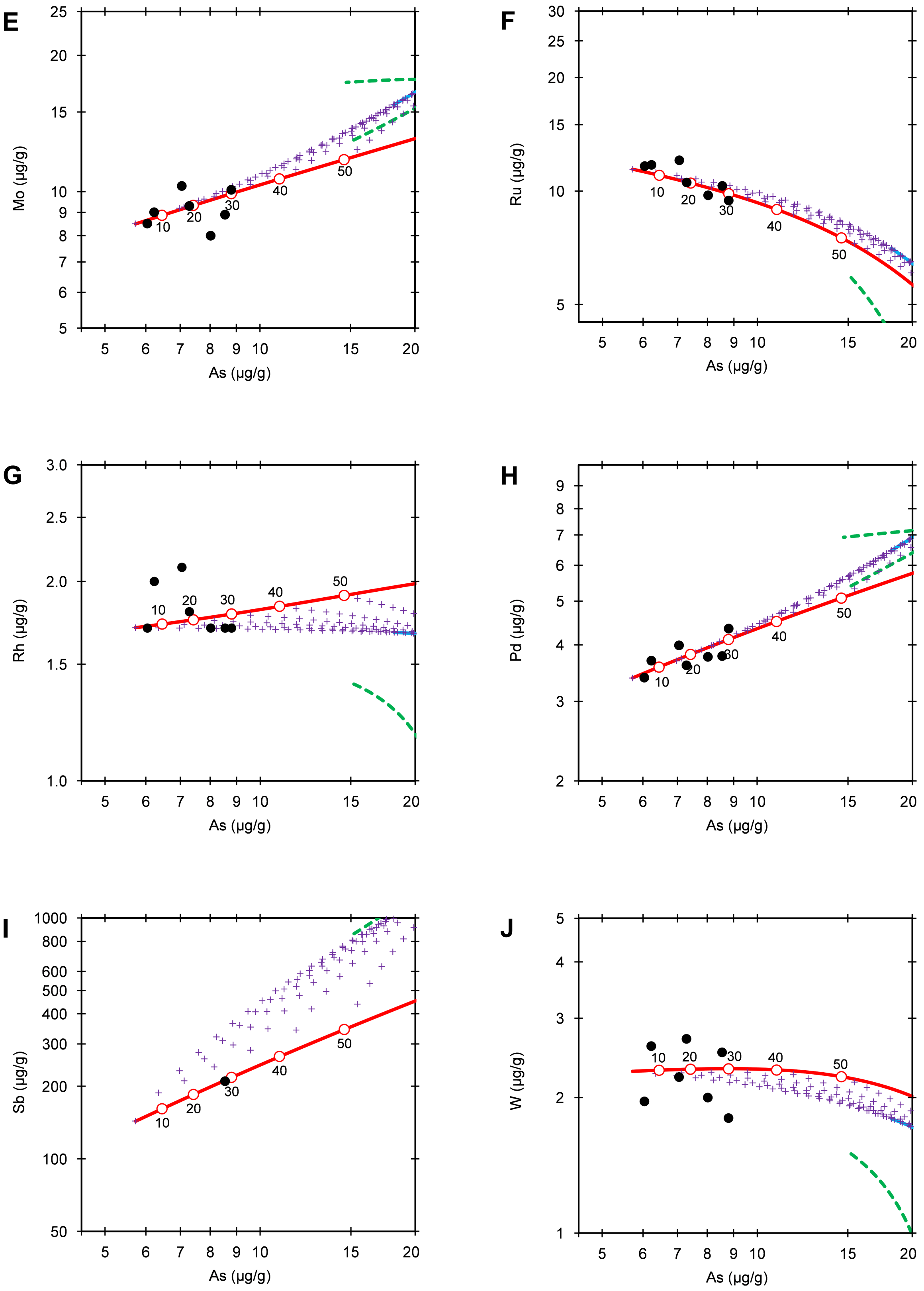
E
Mo (µg/g)
25
20
15
10
9
8
7
6
5
10
20
30
40
50
As (µg/g)
F
Ru (µg/g)
30
25
20
15
10
5
10
20
30
40
50
As (µg/g)
G
Rh (µg/g)
3.0
2.5
2.0
1.5
1.0
10
20
30
40
50
As (µg/g)
H
Pd (µg/g)
9
8
7
6
5
4
3
2
10
20
30
40
50
As (µg/g)
I
Sb (µg/g)
1000
800
600
500
400
300
200
100
50
10
20
30
40
50
As (µg/g)
J
W (µg/g)
5
4
3
2
1
10
20
30
40
50
As (µg/g)
5 6 7 8 9 10 15 20

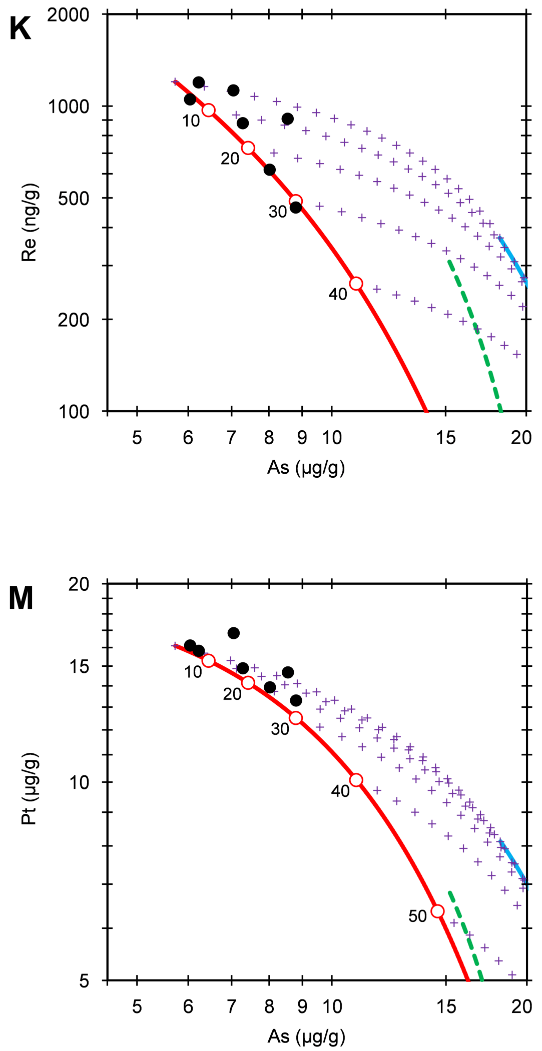

Fig. S2. Crystallization modeling of siderophile elements in group IIC. The model uses bulk 6 wt % S and 2.2 wt % P. The black dots are the NAA data. The red lines, blue lines, and green dashed lines denote the solid resulting from simple fractional crystallization (*SFC solid*), solid from trapped melt (*TM solid*), and liquid (*Liquid*), respectively. The purple crosses are the mixing lines (*Mixing line*) between fractional-crystallization and trapped-melt solids at an increment of 5%. The labeled circles on the red lines represent the crystallization sequence (*Cryst.%*).

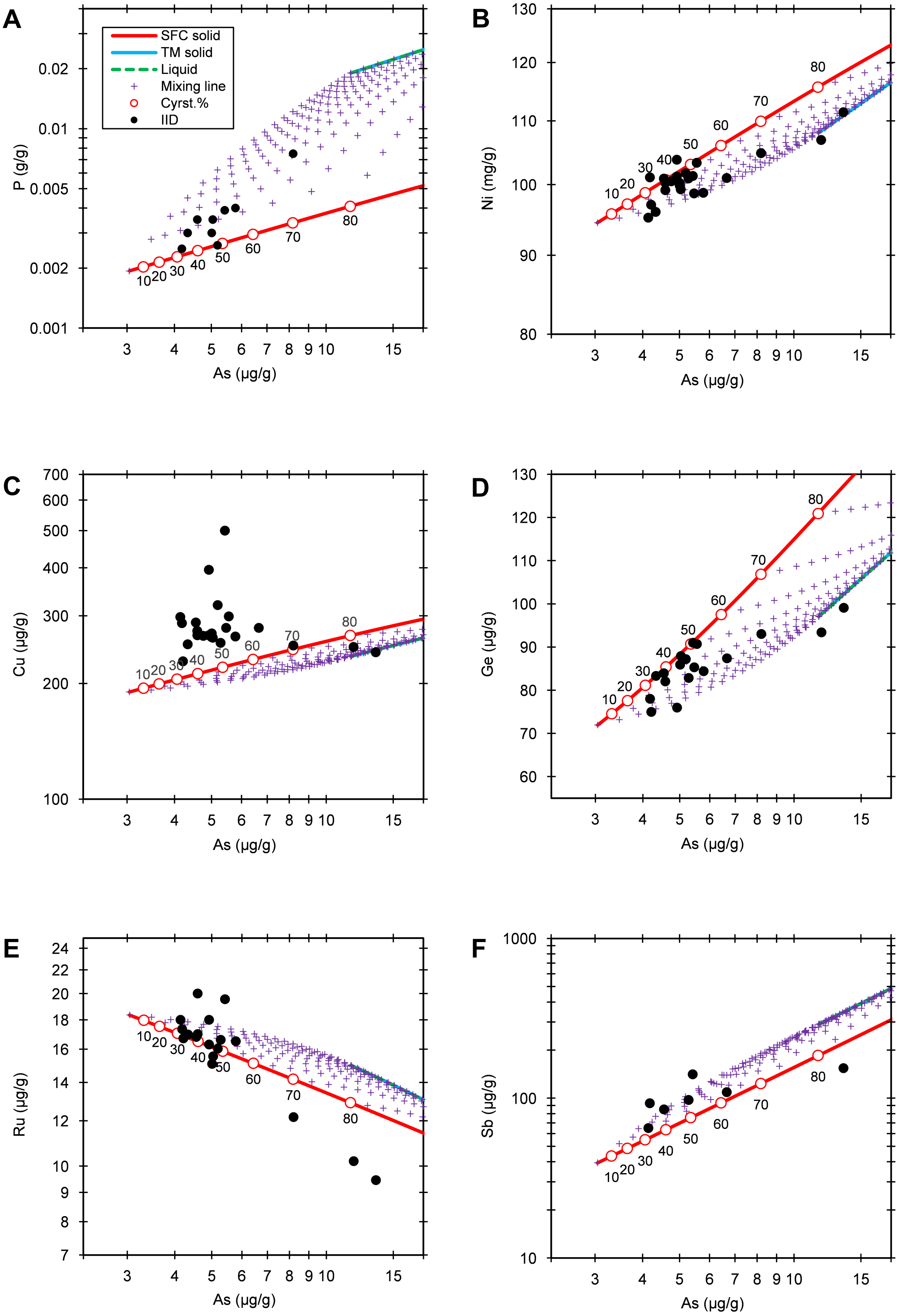

A
B
C
D
E
F
SFC solid
TM solid
Liquid
Mixing line
Cyrst.%
IID
P (g/g)
Ni (mg/g)
Cu (µg/g)
Ge (µg/g)
Ru (µg/g)
Sb (µg/g)
As (µg/g)

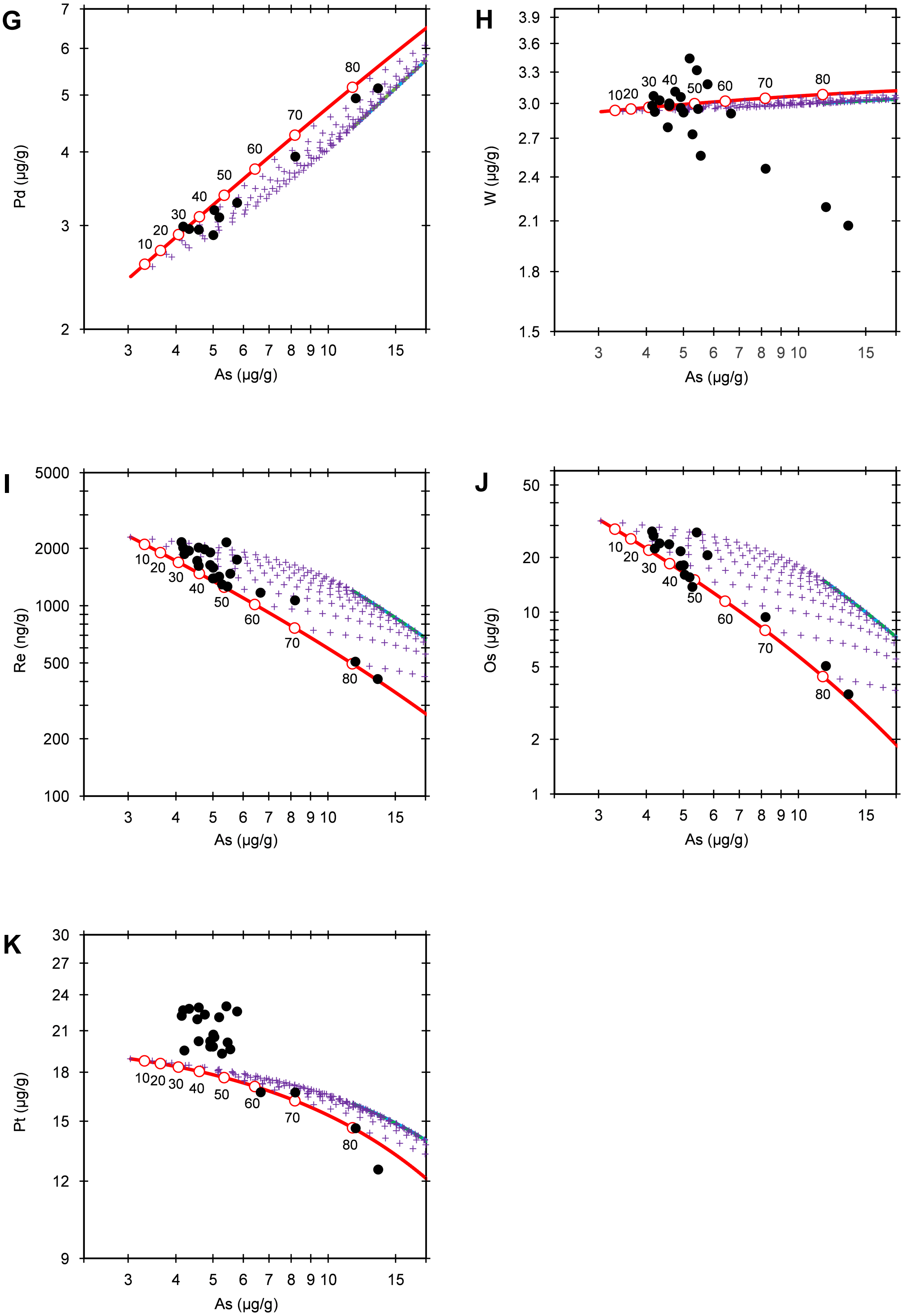
G
Pd (µg/g)
As (µg/g)
H
W (µg/g)
As (µg/g)
I
Re (ng/g)
As (µg/g)
J
Os (µg/g)
As (µg/g)
K
Pt (µg/g)
As (µg/g)

Fig. S3. Crystallization modeling of siderophile elements in group IID. The model uses bulk 0.01 wt % S and 1.9 wt % P. The black dots are the NAA data. The red lines, blue lines, and green dashed lines denote the solid resulting from simple fractional crystallization (*SFC solid*), solid from trapped melt (*TM solid*), and liquid (*Liquid*), respectively. The purple crosses are the mixing lines (*Mixing line*) between fractional-crystallization and trapped-melt solids at an increment of 5%. The labeled circles on the red lines represent the crystallization sequence (*Cryst.%*). The liquid and trapped-melt tracks overlap due to the low S content.

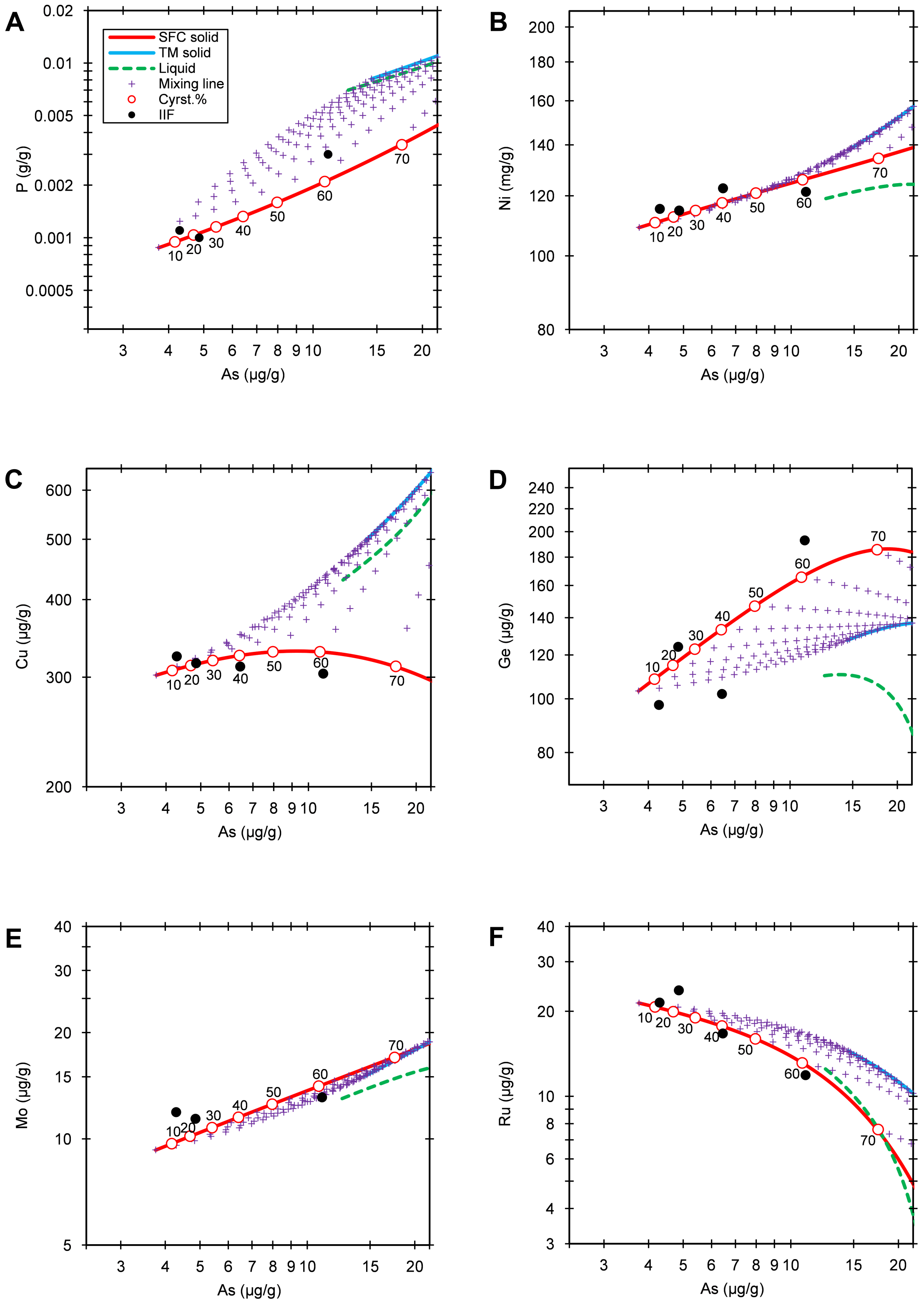

A
SFC solid
TM solid
Liquid
Mixing line
Cyrst.%
IIF
P (g/g)
As (µg/g)
B
Ni (mg/g)
As (µg/g)
C
Cu (µg/g)
As (µg/g)
D
Ge (µg/g)
As (µg/g)
E
Mo (µg/g)
As (µg/g)
F
Ru (µg/g)
As (µg/g)

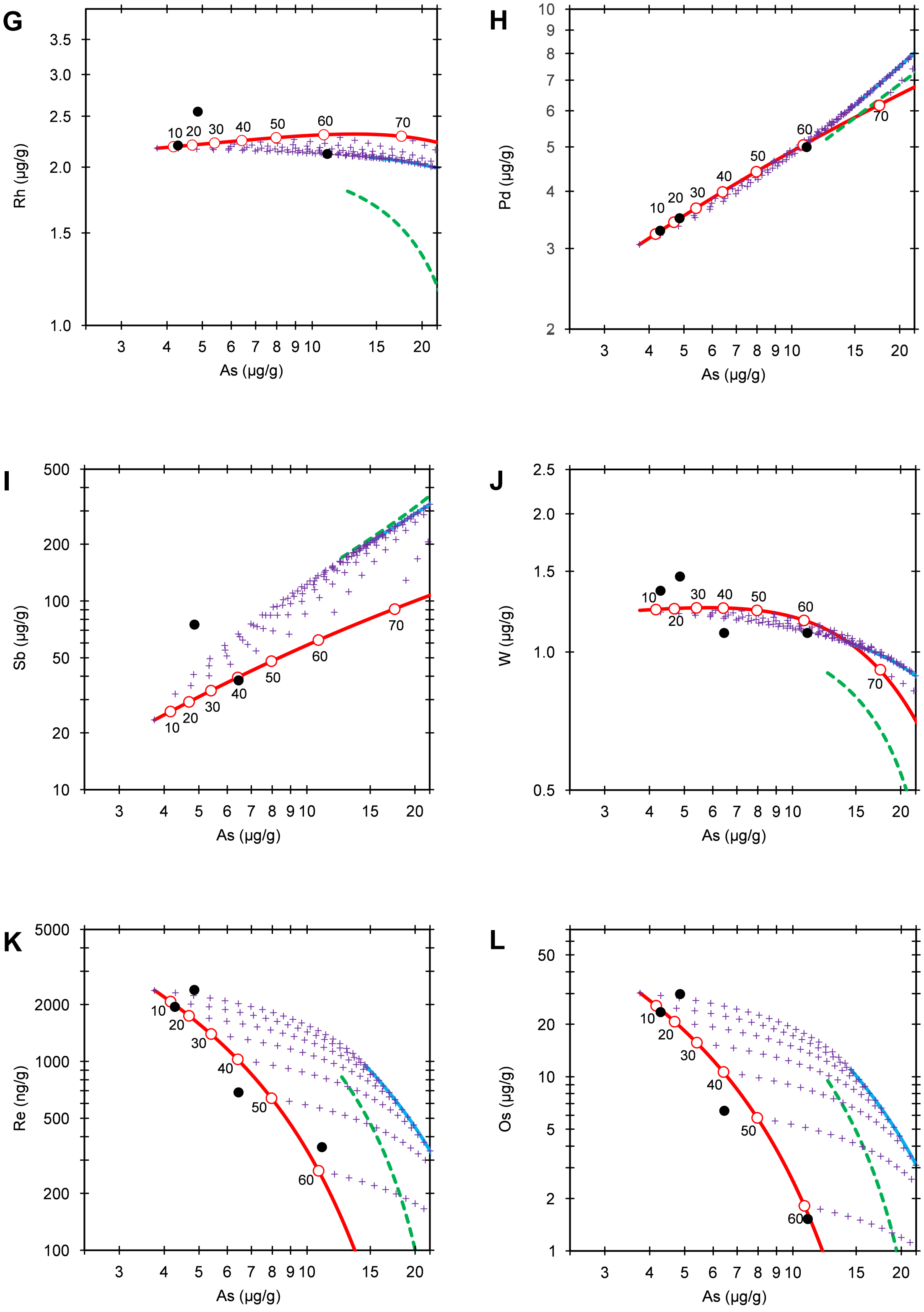
G
Rh (µg/g)
3.5
3.0
2.5
2.0
1.5
1.0
10 20 30 40 50 60 70
3 4 5 6 7 8 9 10 15 20
As (µg/g)
H
Pd (µg/g)
10
9
8
7
6
5
4
3
2
10 20 30 40 50 60 70
3 4 5 6 7 8 9 10 15 20
As (µg/g)
I
Sb (µg/g)
500
200
100
50
20
10
10 20 30 40 50 60 70
3 4 5 6 7 8 9 10 15 20
As (µg/g)
J
W (µg/g)
2.5
2.0
1.5
1.0
0.5
10 20 30 40 50 60 70
3 4 5 6 7 8 9 10 15 20
As (µg/g)
K
Re (ng/g)
5000
2000
1000
500
200
100
10 20 30 40 50 60
3 4 5 6 7 8 9 10 15 20
As (µg/g)
L
Os (µg/g)
50
20
10
5
2
1
10 20 30 40 50 60
3 4 5 6 7 8 9 10 15 20
As (µg/g)

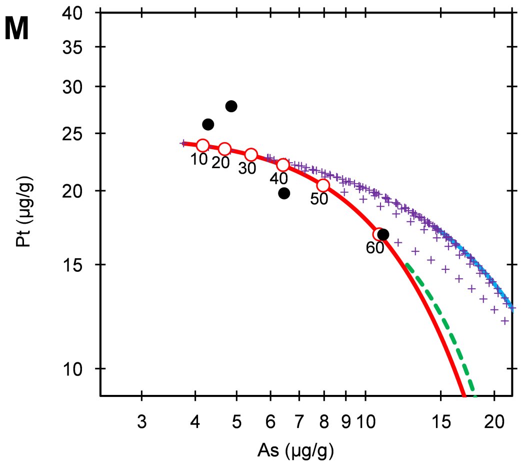

Fig. S4. Crystallization modeling of siderophile elements in group IIF. The model uses bulk 5 wt % S and 0.70 wt % P. The black dots are the NAA data. The red lines, blue lines, and green dashed lines denote the solid resulting from simple fractional crystallization (*SFC solid*), solid from trapped melt (*TM solid*), and liquid (*Liquid*), respectively. The purple crosses are the mixing lines (*Mixing line*) between fractional-crystallization and trapped-melt solids at an increment of 5%. The labeled circles on the red lines represent the crystallization sequence (*Cryst.%*).

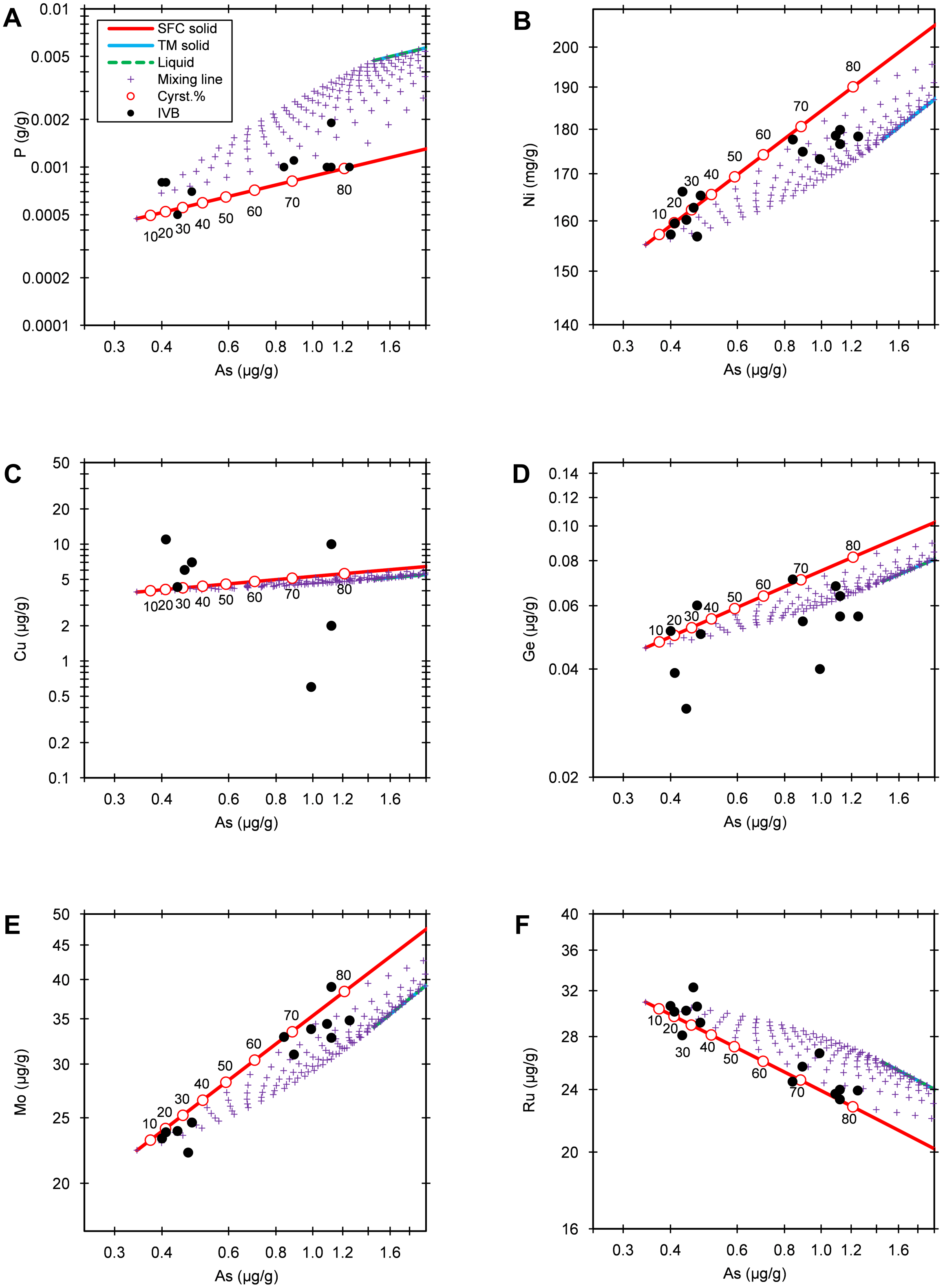

A
B
C
D
E
F
SFC solid
TM solid
Liquid
Mixing line
Cyrst.%
IVB
P (g/g)
Ni (mg/g)
Cu (µg/g)
Ge (µg/g)
Mo (µg/g)
Ru (µg/g)
As (µg/g)
0.3
0.4
0.6
0.8
1.0
1.2
1.6
0.01
0.005
0.002
0.001
0.0005
0.0002
0.0001
200
190
180
170
160
150
140
50
20
10
5
2
1
0.5
0.2
0.1
0.14
0.12
0.10
0.08
0.06
0.04
0.02
45
40
35
30
25
40
36
32
28
24
16
10
20
30
40
50
60
70
80

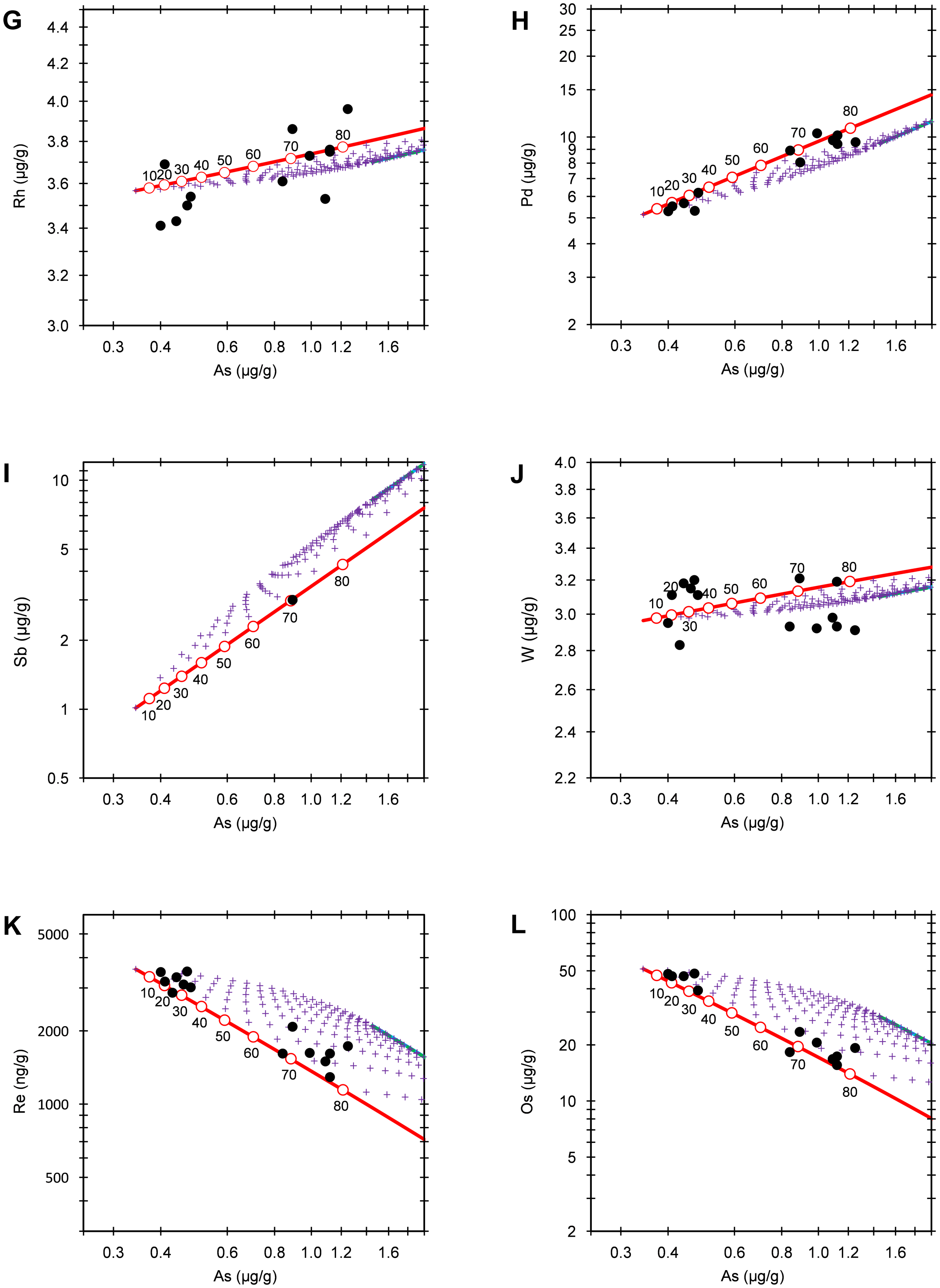
G
Rh (µg/g)
As (µg/g)
H
Pd (µg/g)
As (µg/g)
I
Sb (µg/g)
As (µg/g)
J
W (µg/g)
As (µg/g)
K
Re (ng/g)
As (µg/g)
L
Os (µg/g)
As (µg/g)
10
20
30
40
50
60
70
80
0.3
0.4
0.6
0.8
1.0
1.2
1.6

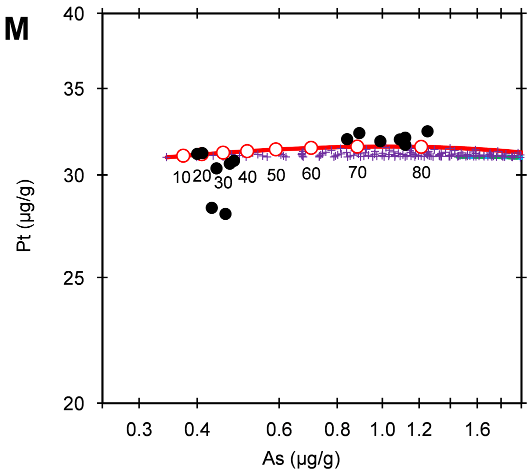

Fig. S5. Crystallization modeling of siderophile elements in group IVB. The model uses bulk 0.01 wt % S and 0.47 wt % P. The black dots are the NAA data. The red lines, blue lines, and green dashed lines denote the solid resulting from simple fractional crystallization (*SFC solid*), solid from trapped melt (*TM solid*), and liquid (*Liquid*), respectively. The purple crosses are the mixing lines (*Mixing line*) between fractional-crystallization and trapped-melt solids at an increment of 5%. The labeled circles on the red lines represent the crystallization sequence (*Cryst.%*). The liquid and trapped-melt tracks overlap due to the low S content.

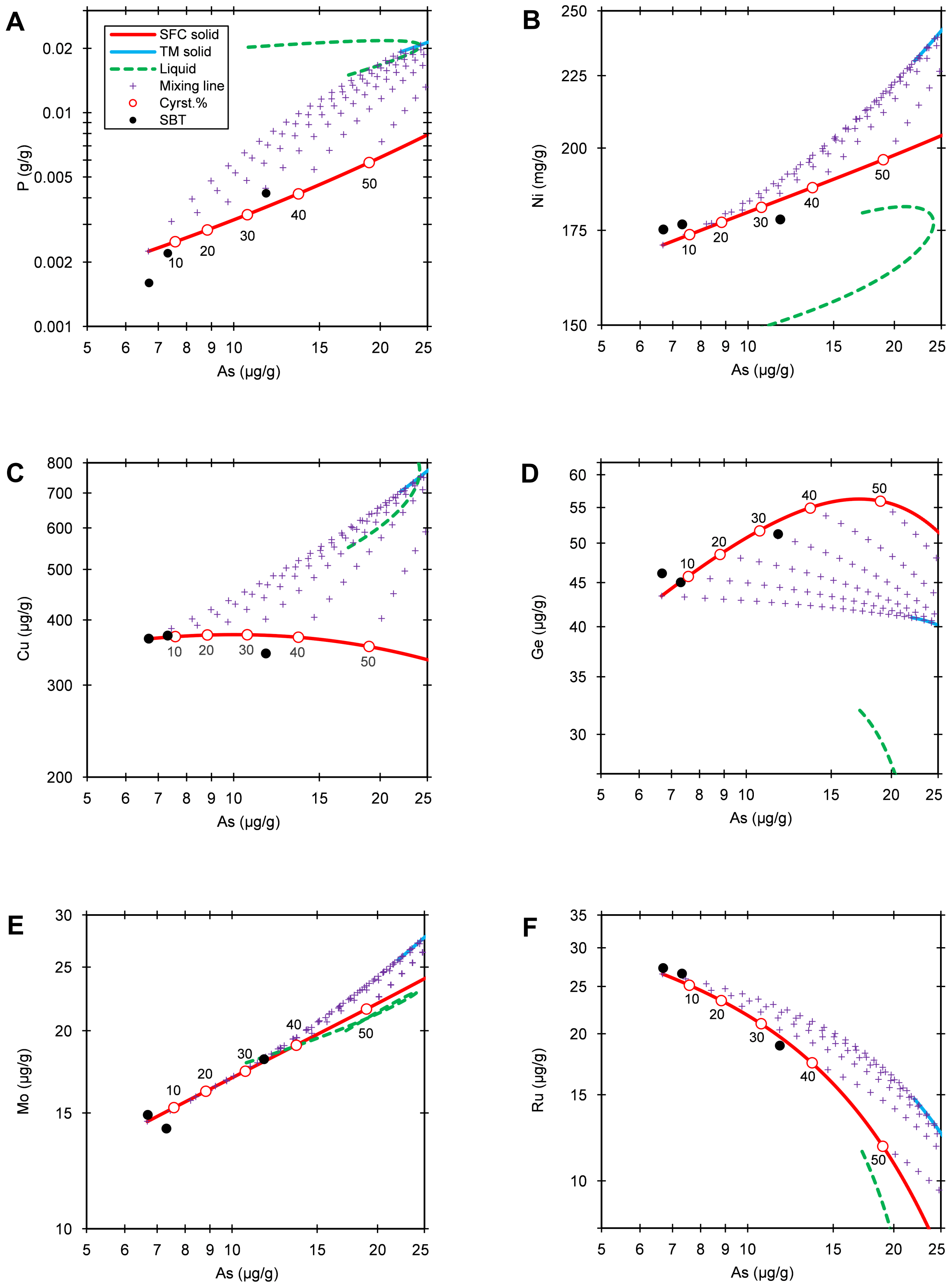

A
SFC solid
TM solid
Liquid
Mixing line
Cyrst.%
SBT
P (g/g)
As (µg/g)
B
Ni (mg/g)
As (µg/g)
C
Cu (µg/g)
As (µg/g)
D
Ge (µg/g)
As (µg/g)
E
Mo (µg/g)
As (µg/g)
F
Ru (µg/g)
As (µg/g)

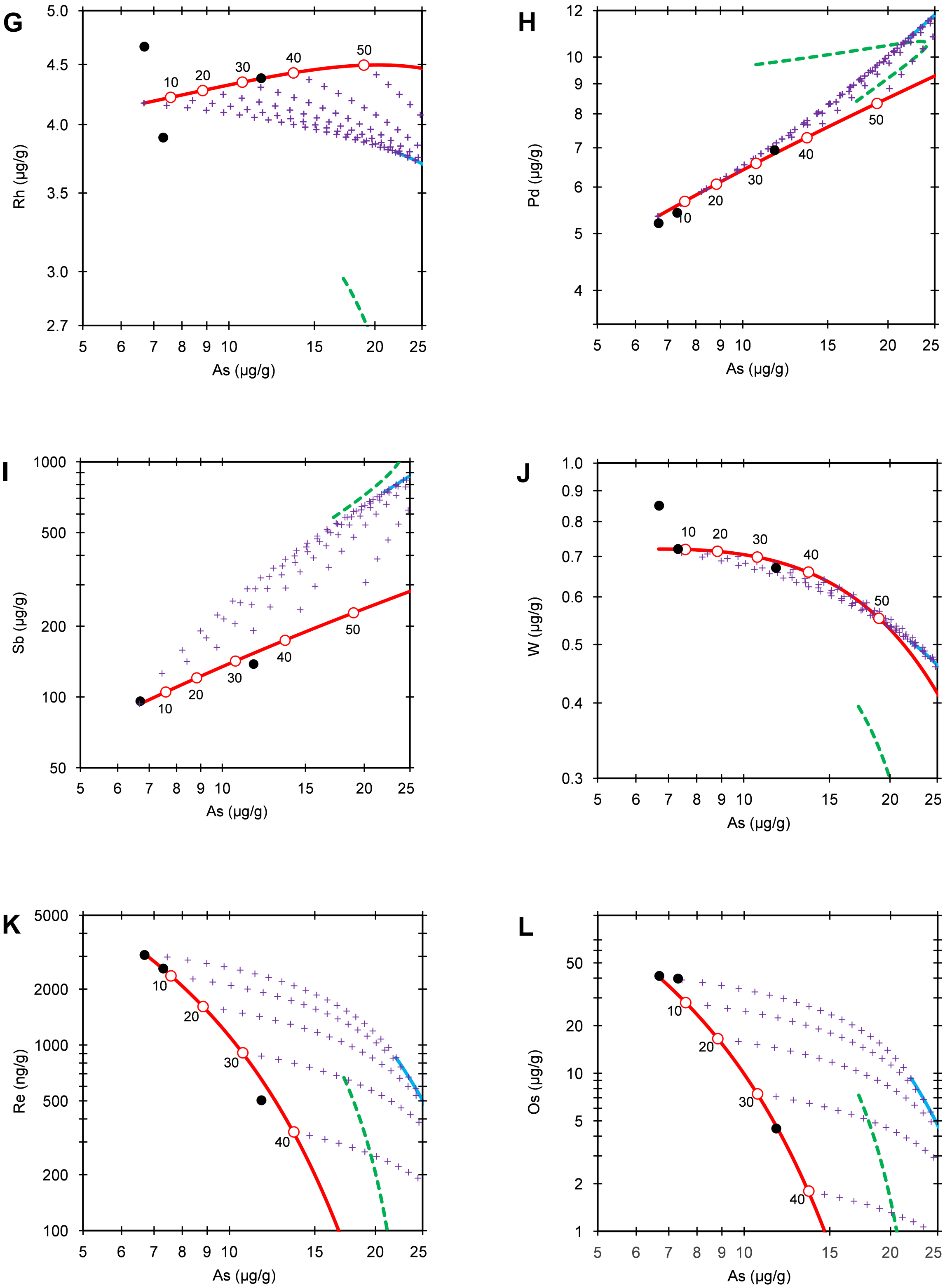
G
Rh (µg/g)
5.0
4.5
4.0
3.5
3.0
2.7
10
20
30
40
50
5 6 7 8 9 10 15 20 25
As (µg/g)
H
Pd (µg/g)
12
10
9
8
7
6
5
4
10
20
30
40
50
5 6 7 8 9 10 15 20 25
As (µg/g)
I
Sb (µg/g)
1000
500
200
100
50
10
20
30
40
50
5 6 7 8 9 10 15 20 25
As (µg/g)
J
W (µg/g)
1.0
0.9
0.8
0.7
0.6
0.5
0.4
0.3
10
20
30
40
50
5 6 7 8 9 10 15 20 25
As (µg/g)
K
Re (ng/g)
5000
2000
1000
500
200
100
10
20
30
40
5 6 7 8 9 10 15 20 25
As (µg/g)
L
Os (µg/g)
50
20
10
5
2
1
10
20
30
40
5 6 7 8 9 10 15 20 25
As (µg/g)

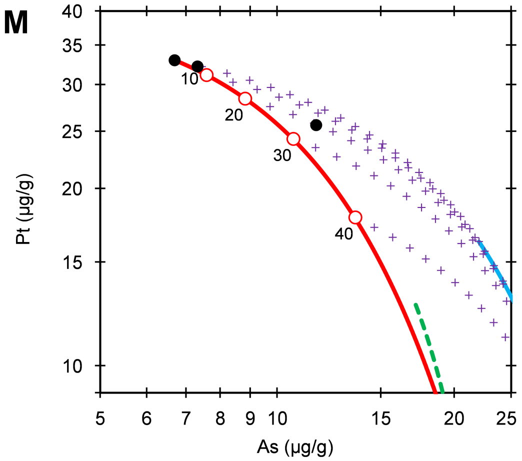

Fig. S6. Crystallization modeling of siderophile elements in the SBT. The model uses bulk 8 wt % S and 1.5 wt % P. The black dots are the NAA data. The red lines, blue lines, and green dashed lines denote the solid resulting from simple fractional crystallization (*SFC solid*), solid from trapped melt (*TM solid*), and liquid (*Liquid*), respectively. The purple crosses are the mixing lines (*Mixing line*) between fractional-crystallization and trapped-melt solids at an increment of 5%. The labeled circles on the red lines represent the crystallization sequence (*Cryst.%*).

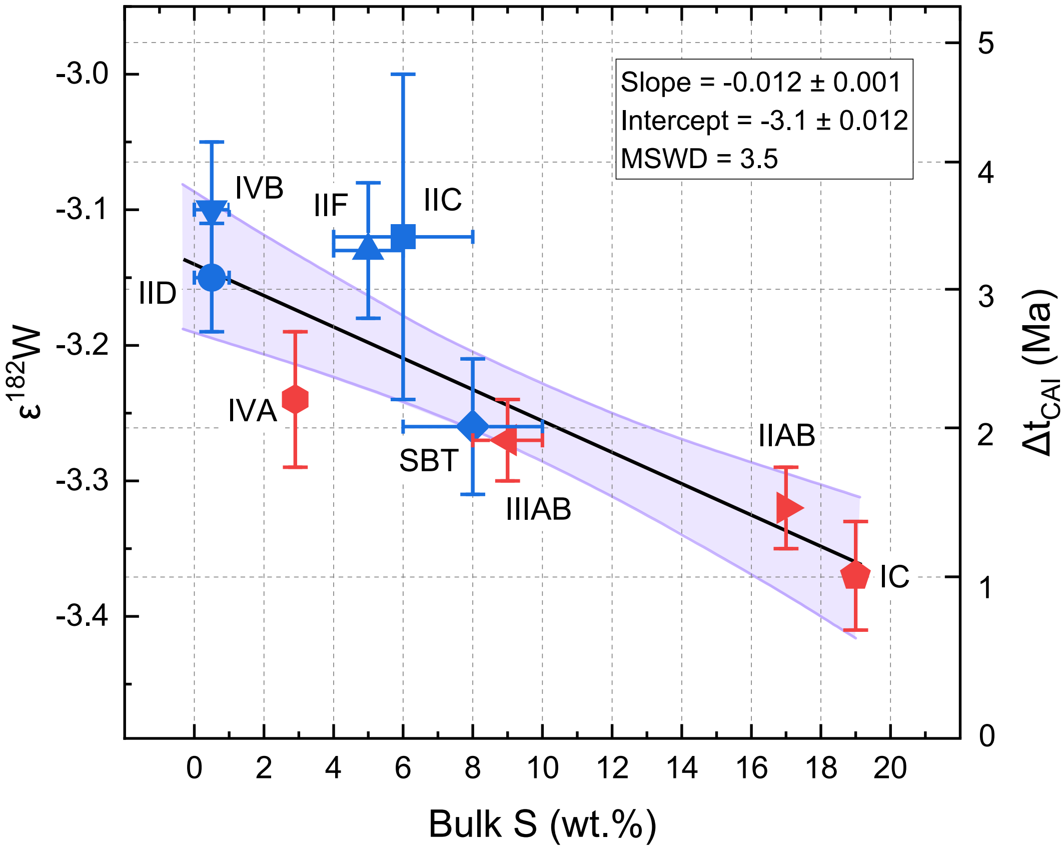

Fig. S7. Bulk S concentrations plotted against ε^{182}W values. All ε^{182}W values and differentiation ages are from the literature (*16*). The bulk S concentrations of NC-iron groups IIC (*29*), IIAB (*26*), IIIAB (*32*), and IVA (*35*) are shown in red symbols, and the CC-iron groups in blue symbols. The solid line is the maximum-likelihood fit with 1σ error envelope for all groups. Error bars on the ε^{182}W values are 95% confidence limits of the means.

Table S1. **Mean compositions of irons in groups IIC, IID, IIF, IVB, and the South Byron Trio (SBT).** The meteorites in each group are arranged in order of increasing As.

Meteorite	Cr (µg/g)	Co (mg/g)	Ni (mg/g)	Cu (µg/g)	Ga (µg/g)	Ge (µg/g)	As (µg/g)	Mo (µg/g)	Ru (µg/g)	Rh (µg/g)	Pd (µg/g)	Sb (ng/g)	W (µg/g)	Re (ng/g)	Os (µg/g)	Ir (µg/g)	Pt (µg/g)	Au (µg/g)	P* (%)
**IIC**																			
Cratheús (1950)	174	5.49	92.7	250	36.5	91.5	6.04	*8.5*	***11.65***	*1.7*	***3.385***	<150	1.96	1051	***13.07***	11.4	13.2†	0.772	0.30
Perryville	151	5.43	93.7	262	38.0	88.0	6.14	*9.0*	***11.73***	*2.0*	***3.689***	220	2.18	1129	13.2†	11.8	16.3†	0.828	0.34
Darinskoe	116	5.54	102.7	290	37.4	114.0	7.05	*10.3*	***12.07***	*2.1*	***3.992***	<150	2.22	1125	***13.74***	12.0	13.4†	0.896	
Kumerina	99	5.52	97.8	282	38.2	93.4	7.29	*9.3*	***10.54***	*1.8*	***3.603***	<120	2.70	879	***9.95***	9.14	13.0†	0.893	0.40
Salt River	85	5.58	100.8	244	37.9	100.0	8.02	*8.0*	***9.74***	*1.7*	***3.765***	<150	2.00	618	***7.01***	6.98	10.8†	1.000	0.43
Ballinoo	102	5.55	98.3	267	36.8	94.4	8.55	*8.9*	***10.32***	*1.7*	***3.778***	420	2.52	908	***10.51***	9.24	15.5†	0.979	0.48
Unter-Mässing	104	5.58	97.8	245	38.2	100.9	8.80	*10.1*	***9.44***	*1.7*	***4.347***	<150	1.80	465	***4.11***	5.08	13.1†	1.072	0.40
**IID**																			
Caçapava do Sul	82	6.69	95.2	298	69.7	<140	4.14		18.0			65	2.98	2160	27.8	21.6	22.2	0.563	
Losttown	**46**	**6.60**	101.1	**287**	**71.9**	**78.0**	4.18		***17.34***		***2.988***	**93**	**3.07**	**2020**	***26.30***	**20.7**	**19.3†**	**0.564**	0.25
GP04	51	6.66	98.7	282	70.5		4.31		17.8			<100	2.97	1977	23.4	18.9	20.9	0.602	
N'Kandhla	**68**	**6.76**	**96.0**	**253**	**71.3**	**83.3**	**4.33**		***16.97***		***2.959***	**<100**	**3.03**	**1942**	***23.87***	**19.6**	**19.9†**	**0.585**	0.30
Alt Bela	**82**	**6.62**	**100.9**	**288**	**73.6**	**83.9**	**4.55**		**16.8**			**85**	**2.79**	**1718**		**16.6**	**21.9**	**0.620**	
Bridgewater	**202**	**6.63**	**99.2**	**274**	**74.9**	**82.0**	**4.59**		***17.00***		***2.950***	**<100**	**2.98**	**2017**	***23.63***	**19.7**	**22.0†**	**0.599**	0.35
Cheder	**54**	**6.62**	**100.3**	**267**	**71.5**	**<168**	**4.59**		**20.0**			**<120**	**3.00**	**1618**		**17.5**	**20.2**	**0.586**	
Saint Augustine	**85**	**6.66**	**100.5**	**266**	**74.7**		**4.76**					**<150**	**3.11**	**1980**		**19.4**	**22.3**	**0.608**	
Nothing	92	6.63	101.2	395	75.2	76.0	4.92		18.0			<150	2.96	1636	18.0	16.0	20.2	0.628	
Northwest Africa 10224	54	6.59	103.8	267	75.5	<120	4.92		16.3			<170	3.06	1904	21.6	17.5	19.8	0.628	
Mafuta	**62**	**6.71**	**100.3**	**264**	**72.7**	**<100**	**5.00**					**<150**	**2.92**	**1390**		**15.4**	**19.8**	**0.645**	
Elbogen	**53**	**6.70**	**99.9**	**270**	**74.3**	**85.9**	**5.01**		***15.08***		***2.890***	**<100**	**2.92**	**1583**	***18.11***	**15.9**	**19.0†**	**0.641**	0.30
Puquios	**48**	**6.67**	**99.3**	**263**	**76.4**	**87.9**	**5.04**		***15.55***		***3.185***	**<100**	**4.04**	**1393**	***16.06***	**14.3**	**19.9†**	**0.655**	0.35
Carbo	**88**	**6.69**	**101.7**	**320**	**77.1**	**87.2**	**5.19**		***16.03***		***3.097***	**<100**	**3.44**	**1421**	***15.54***	**14.3**	**22.4†**	**0.671**	0.26
Gheriat 004	52	6.81	100.9	255	71.2	83.0	5.28		16.6			97	2.73	1295	13.8	13.1	19.3	0.702	
Richa	137	6.77	101.3	500	80.3	91.0	5.42		19.6			141	3.32	2152	27.4	20.7	23.0	0.670	0.39
Brownfield (iron)	**46**	**6.73**	**98.7**	**279**	**75.4**	**85.3**	**5.46**					**<100**	**2.95**	**1260**		**12.9**	**20.1**	**0.722**	
Hraschina	**77**	**6.89**	**103.3**	**299**	**77.0**	**90.7**	**5.55**					**<130**	**2.56**	**1472**		**14.6**	**19.6**	**0.700**	
Mount Ouray	**86**	**6.69**	**98.8**	**265**	**73.9**	**84.4**	**5.78**		***16.51***		***3.280***	**<100**	**3.18**	**1744**	***20.59***	**17.8**	**23.0†**	**0.700**	0.40
Vicenice	**61**	**6.75**	**101.0**	**279**	**76.1**	**87.4**	**6.66**					**109**	**2.91**	**1170**		**12.6**	**16.7**	**0.762**	
Rodeo	**138**	**6.88**	**104.8**	**251**	**76.5**	**93.0**	**8.20**		***12.18***		***3.930***	**<100**	**2.46**	**1066**	***9.38***	**10.2**	**15.5†**	**0.947**	0.75
Needles	**31**	**7.01**	**106.9**	**249**	**85.6**	**93.4**	**11.8**		***10.20***		***4.935***	**<130**	**2.19**	**508**	***5.06***	**5.37**	**13.3†**	**1.404**	~0.85
Wallapai	**32**	**6.91**	**111.4**	**241**	**84.3**	**99.1**	**13.5**		***9.449***		***5.133***	**154**	**2.07**	**412**	***3.53***	**3.99**	**11.6†**	**1.533**	~0.90
**IID-an**																			
Arltunga	**121**	**6.40**	**96.6**	**264**	**71.7**	**83.0**	**4.06**		**19.8**		***2.868***	**<150**	**2.86**	**2070**	***26.26***	**20.6**	**21.6**	**0.559**	

Northeast Africa 002	**132**	**6.58**	**102.2**	**259**	**70.6**		**4.25**		**20.3**		***2.774***	*<150*	*3.01*	*2150*	***28.74***	*22.6*	*22.4*	*0.556*	
Los Vientos 189	19	6.60	106.2	241	61.1	96.0	4.90		17.2			<150	2.96	1954	23.2	18.8	22.0	0.504	
**IIF**																			
Del Rio	114	6.75	115.3	324	8.25	97.4	4.29	*11.9*	***21.49***	*2.2*	***3.282***	<200	1.36	1942	***23.47***	20.7	22.6†	0.564	0.11
Dorofeevka	41	6.84	114.7	316	8.60	124.0	4.86	*11.4*	***23.75***	*2.55*	***3.494***	75	1.46	2390	***29.82***	24.9	26.6†	0.635	0.10
Northwest Africa 6932	45	6.84	122.7	312	9.45	102	6.45		16.7			38	1.10	685	6.4	7.87	19.8	0.784	
Repeev Khutor	78	6.92	121.4	304	11.0	193	11.0	*13.1*	***11.88***	*2.12*	***4.997***	<300	1.10	352	***1.525***	3.03	***16.86***	1.119	0.30
**IIF-an**																			
Purmela	86	6.05	114.4	307	12.9	<150	4.17	*10.4*	***13.85***	*1.60*	***3.484***	<200	0.88	1030	***10.99***	11.0	18.7	0.511	
Monahans (1938)	129	6.02	105.4	311	8.84	123	4.52	*13*	***20.77***	*2.34*	***3.229***	<150	1.93	1207	***12.02***	14.3	22.9	0.584	0.09
Corowa	28	6.50	134.9	280	10.3	159	17.5	*17*	***3.33***	*1.48*	***6.339***	<180	0.51	110	***0.80***	0.877	4.8	1.827	0.30
**IVB**																			
Iquique	317	7.57	157.2	<5	0.170	0.051	0.400	*23.3*	26.5†	3.41§	***5.287***	<200	2.95	3490	50.3†	30.9	28.1†	0.062	0.08
Kokomo	268	7.54	159.5	11	0.193	0.039	0.410	*23.8*	31.0†	3.69§	***5.514***		3.11	3185	***46.75***	28.9	23.5†	0.063	0.08
Catalina 003	249	7.60	166.1	~3	0.170	<20	0.430		28.1			<150	2.83	2868	27.4	27.6	28.3	0.070	
Cape of Good Hope	287	7.68	156.8	<6	0.189	0.060	0.470	*22.2*	27.1†	3.50§	***5.306***	<150	3.20	3503	45.1†	27.1	26.5†	0.054	
Tlacotopec	257	7.61	160.2	4.3	0.195	0.031	0.44	*23.9*	***30.19***	3.43§	***5.665***		3.18	3320	***46.77***	29.7	27.2†	0.063	0.05
Dumont	340	7.64	162.7	6	0.200	<50	0.460		32.3			<150	3.15	3100		28.5	28.0	0.063	
Hoba	216	7.64	165.3	7	0.192	0.050	0.480	*24.6*	31.6†	3.54§	***6.198***	<180	3.11	3017	***39.22***	27.1	27.1†	0.077	0.07
Weaver Mountains	116	7.80	177.6	<5	0.232	0.071	0.84	*32.9*	***24.55***	3.61§	***8.906***		2.93	1613	***18.28***	16.2	28.7†	0.121	0.10
Santa Clara	121	7.85	174.9	<5.4	0.220	0.054	0.893	*31.0*	***25.65***	3.86§	***8.055***	3.0	3.21	2077	***23.52***	19.2	27.7†	0.110	0.11
Tinnie	101	7.96	173.2	0.6	0.258	*0.04*	0.99	*33.8*	***26.66***	3.73§	***10.32***	<200	2.92	1621	***20.57***	16.4	29.8†	0.135	
Tawallah Valley	85	7.80	178.5	<7	0.200	0.068	1.09	*34.4*	20.8†	3.53§	***9.721***	<230	2.98	1496	18.5†	15.5	25.3†	0.154	0.10
Skookum	45	7.92	176.6	2	0.272	0.056	1.12	*32.8*	27.0†	3.76§	***9.420***	<150	2.93	1614	***17.34***	16.1	28.2†	0.144	0.19
Warburton Range	82	7.84	179.9	10.0	0.244	0.064	1.12	*39*	25.8†	3.75§	***10.14***	<150	3.19	1291	***15.59***	13.9	31.2†	0.164	0.10
Ternera [Galleguillos]	71	7.88	176.3	4	0.253	0.063	1.16						2.87	1676		17.2		0.155	0.10
Ternera	87	7.86	178.3	<1	0.261	0.056	1.25	*34.8*	27.6†	3.96§	***9.567***	<190	2.91	1730	***19.27***	16.9	27.3†	0.147	0.10
**South Byron Trio**																			
Babb's Mill (Troost's Iron)	71	**9.14**	**176.9**	**372**	**17.5**	***46.1***	**6.52**	*14.9*	***27.25***	*4.66*	***5.205***		1.33‡	2806	***41.29***	**32.2**	***32.99***	**0.982**	0.16
South Byron	67	**9.19**	**176.7**	**373**	**18.6**	**45.0**	**7.32**	*14.2*	***26.55***	*3.9*	***5.422***		1.24‡	2578	***39.74***	**30.4**	***32.20***	**1.120**	0.22
Inland Forts 83500	30	**9.64**	**178.1**	**345**	**19.7**	***51.2***	**11.7**	*18.1*	***18.91***	*4.38*	***6.934***	122	0.71‡	504	***4.47***	**7.19**	***25.61***	**1.592**	0.42

Values in italics, bold text, and bold italics are LA-ICP-MS, NAA, and ID-ICP-MS data, respectively, from the literature: IIC (*21*), IID (*22, 23*), IIF (*24*), IVB (*27*), and SBT (*71*, *28, 43*).
*P concentrations are from modal analysis (*70*).
†INAA data are reported here but ID-ICP-MS data from the literature are used in the models: IIC (*21*), IID (*22, 23*), IIF (*24*), and IVB (*27*).
‡INAA data are reported here but LA-ICP-MS data from the literature (*43*) are used in the model.
§Rh data of group IVB are from standard addition ICP-MS (*27*).

Table S2. Replicate NAA data for groups IIC, IID, IIF, IVB, and the SBT irons.

Meteorite	Date	Mass (mg)	Cr (µg/g)	Co (mg/g)	Ni (mg/g)	Cu (µg/g)	Ga (µg/g)	Ge (µg/g)	As (µg/g)	Ru (µg/g)	Sb (ng/g)	W (µg/g)	Re (ng/g)	Os (µg/g)	Ir (µg/g)	Pt (µg/g)	Au (µg/g)
**IIC**																	
Ballinoo	91/05	454	98	5.55	101.5	267	34.5		8.67			2.42	825		9.17	13.3	0.959
Ballinoo	96/04	466	106	5.54	96.1	267	36.9		8.43		420	2.62	990		9.31	17.7	0.998
Cratheús (1950)	91/05	610	132	5.48	98.0	253	32.0		6.11			1.98	1010		11.3	12.7	0.760
Cratheús (1950)	99/08	555	216	5.49	90.2	247	38.9		5.97		95	1.93	1092		11.4	13.7	0.783
Darinskoe	03/09	632	120	5.47	105.5	284	39.1	114.0	7.19		<100	2.24	1175		12.0	13.6	0.910
Darinskoe	04/04	574	113	5.62	99.9	296	35.6	<180	6.76		<150	2.20	1074		12.0	13.2	0.882
Kumerina	79/09	673	88	5.35	97.9	282	38.5		7.20		241	4.25	790		9.22		0.940
Kumerina	84/03	338	89	5.82	95.6	286	37.7		7.44			1.74	800		9.00		0.919
Kumerina	97/09	-	120	5.45	99.2	280	39.0		7.26		<120	2.41	963		9.17	13.0	0.870
Perryville	79/09	421	138	5.24	93.1	263	38.9		6.40		<650	2.30	1150		11.9		0.860
Perryville	80/06	453		5.44	93.7	273	38.6		6.17			2.26	1069		12.0		0.831
Perryville	04/11	517	142	5.42	91.0	254	37.9		5.84	17.4	325	2.03	1038		11.6	17.2	0.817
Perryville	18/02	407	165	5.53	98.2	258	37.5		6.30	11.5	186	2.13	1258	13.2	11.6	15.5	0.803
Salt River	79/09	481	93	5.50	101.5	256	39.9		8.50		<440	2.14	601		7.30		1.080
Salt River	80/06	358		5.66	102.2	255	37.4		8.05			1.85	585		7.14		1.002
Salt River	99/09	498	81	5.54	100.3	234	36.5		7.53		<150	1.77	609		6.95	10.8	0.960
Unter-Mässing	99/08	575	104	5.60	96.2	248	38.0		8.87		123	1.81	463		5.03	12.5	1.066
Unter-Mässing	99/09	539	103	5.56	99.3	241	38.4		8.73		<150	1.79	467		5.12	13.7	1.077
**IID**																	
Caçapava do Sul	17/08	557	90	6.63	94.3	307	70.8	136	4.29	19.3	41	2.84	2220	26.2	21.6	22.5	0.562
Caçapava do Sul	17/11	467	73	6.75	96.0	290	68.6	<190	4.00	16.8	89	3.13	2099	29.4	21.7	22.0	0.566
Gheriat 004	18/10	514	52	6.81	100.9	255	71.2	83	5.28	16.6	97	2.73	1295	13.8	13.1	19.3	0.702
GP04	21/04	403	51	6.66	98.7	282	70.5	115	4.31	17.8	<100	2.97	1977	23.4	18.9	20.9	0.602
Nothing	13/04	613	149	6.58	103.2	265	77.0	<170	4.96	12.9	<150	2.97	1614	17.4	15.7	19.9	0.620
Nothing	13/07	501	73	6.67	99.3	324	73.5	76	4.87	15.8	<170	2.96	1658	18.8	16.4	20.4	0.636
Northwest Africa 10224	16/10	531	30	6.61	102.9	277	76.0	96	4.89	16.8	<200	3.06	1938	22.1	17.4	20.3	0.624
Northwest Africa 10224	17/04	598	124	6.57	104.8	256	75.0	<130	4.94	15.8	<170	3.05	1871	21.2	17.6	19.3	0.632
Richa	11/05	526	108	6.71	100.8	567	76.6		4.96	18.3	<83	3.06	2041	28.5	19.6	20.7	0.612
Richa	12/02	473	165	6.82	103.0	432	86.4		5.88	20.8	141	3.57	2262	26.3	21.8	25.2	0.727
**IID-an**																	
Los Vientos 189	17/04	521	16	6.50	109.3	240	61.1	132	5.04	17.0	<150	2.95	1939	22.6	18.8	23.7	0.498
Los Vientos 189	17/11	517	22	6.70	103.0	241	61.1	83	4.77	17.4	<330	2.98	1970	23.8	18.8	20.2	0.511
**IIF**																	
Del Rio	78/04	431	90	6.76	116.0	327	8.50		4.40		160	1.40	1900		17.8		0.600

Del Rio	78/06	664	<62	6.77	116.5	325	8.14		4.18		<269	1.22	1909		21.7		0.619
Del Rio	99/09	565	138	6.71	115.4	321	8.18		4.28		<200	1.40	1980		20.9	22.6	0.564
Dorofeevka	78/04	229	<13	6.68	116.0	311	8.55		5.00		<140	1.50	2200		20.3		0.610
Dorofeevka	78/06	440	<67	6.61	116.1	332	8.46		5.31		<503	1.96	2600		25.0		0.635
Dorofeevka	07/06	440	41	6.93	114.0	310	8.56		4.73	27.2	75	1.40	2392		25.5	26.6	0.639
Northwest Africa 6932			45	6.84	122.7	312	9.45	102	6.45	16.7	38	1.10	685	6.4	7.87	19.8	0.784
Repeev Khutor	78/04	247	<51	6.68	145.0	440	11.8		12.9		360	1.63	260		2.58		1.600
Repeev Khutor	78/06	353	<70	6.72	139.0	396	12.4		11.9		199	1.45	<700		3.16		1.348
Repeev Khutor	85/06	198	134	6.92	121.4	304	11.0		11.0		551	1.10	352		3.03		1.119
**IIF-an**																	
Corowa	78/04	383	<23	6.73	137.0	307	10.4		17.6		280	0.50	110		0.790		1.880
Corowa	78/06	460	<29	6.69	131.7	285	9.6		17.1		<178	0.55	<350		0.897		1.791
Corowa	04/12	515	28	6.29	136.4	270	10.5		17.6		<450	0.50	100		0.911	4.8	1.822
Monahans (1938)	78/04	465	131	6.05	107.0	310	8.90		4.50		<170	1.94	1200		12.1		0.600
Monahans (1938)	78/06	371	<57	6.04	107.0	345	8.85		4.67		<268	2.08	1211		15.2		0.619
Monahans (1938)	99/09	572	127	5.99	103.6	295	8.70		4.39		<150	1.77	1210		14.4	22.9	0.567
Purmela	98/08	597	88	6.01	113.3	309	13.3	<130	4.18		<150	0.91	1097		11.1	19.7	0.513
Purmela	99/02	590	85	6.08	115.4	304	12.4	<180	4.16		<200	0.85	959		10.9	17.6	0.508
**IVB**																	
Cape of Good Hope	83/03	648	225	7.58	152.3	<5	<0.22		0.41			3.38	3589		27.1		0.057
Cape of Good Hope	83/05	525	244	7.51	161.4	<25	<0.6		0.48			3.18	3227		23.5		0.054
Cape of Good Hope	13/04	431	393	7.81	156.7	<6	0.160	<125	0.52	27.1	<190	3.03	3693	45.1	30.6	26.5	0.051
Catalina 003	10/08	-	246	7.57	167.4	<2	0.130	<21	0.45	27.0	<150	2.97	2970	35.5	27.2	28.8	0.070
Catalina 003	10/09	-	252	7.62	164.7	3.8	0.220	<33	0.41	29.1	<150	2.68	2765	39.3	27.9	27.8	0.070
Dumont	06/03	550	340	7.64	162.7	6	0.220	<60	0.46	32.3	<150	3.15	3102		28.5	28.5	0.063
Dumont	06/05	535	340	7.64	162.7	6	0.200	<50	0.46	32.3	<150	3.15	3100		28.5	28.0	0.063
Hoba	82/05	612	241	7.57	161.9	<10	<0.4		0.49			2.80	3053		26.9		0.079
Hoba	82/10	499	173	7.72	161.4	8	<0.4		0.580			3.30	2970		27.0		0.079
Hoba	05/08	500	234	7.64	166.6	6	<0.59	<80	0.460	31.6	<180	3.17	3023		27.2	27.1	0.075
Iquique	82/05	728	331	7.56	156.7	<20	<0.4		0.37			4.18	3566		31.1		0.066
Iquique	99/06	533	353	7.59	160.4	3.7	0.200	<50	0.40		<200	2.99	3530		31.1	30.5	0.055
Iquique	12/10	534	272	7.56	151.3	<5	0.460	<50	0.43	26.5	<290	2.91	3375	50.3	30.4	25.7	0.068
Kokomo	77/05	494	230	7.50	161.1	29	<1.73		0.400			2.98	3150		26.1		0.075
Kokomo	78/07	557	302	7.63	157.1	<12	<0.97		0.391			3.16	3559		30.0		0.062
Kokomo	04/12	500	269	7.51	160.3	10.6	<0.43	<209	0.431	30.6	<140	3.15	3015		29.8	24	0.061
Santa Clara	77/05	515	117	7.80	175.6	19	<1.8		0.740			3.06	1730		17.1		0.106
Santa Clara	78/07	520	126	7.78	176.8	<9	<0.96		0.895			3.18	2317		20.1		0.110
Santa Clara	98/04	543	130	7.80	170.0	<5.4	0.400	<50	1.02		<100				18.8	28.6	0.110
Santa Clara	98/06	543	111	7.97	175.3	<5.5	0.580	<50	0.84		<100	3.30	2130		19.5	26.8	0.110
Skookum	04/04	524	55	8.02	177.4	<4	0.041	<51	1.07	27.4	<150	2.92	1560		16.0	27.8	0.145

Skookum	04/07	536	35	7.82	178.5	2	<0.28	<75	1.16	26.5	<150	2.93	1668		16.2	28.6	0.142
Tawallah Valley	82/05	610	100	7.83	178.3	<20	<0.4		1.08			2.69	1539		15.9		0.160
Tawallah Valley	82/10	557	83	7.92	177.3	7	<0.2		1.15			3.27	1528		15.3		0.150
Tawallah Valley	12/10	656	73	7.73	182.2	<7	0.720*	<50	1.05	20.8	<230	4.56*	1422	18.5	15.4	25.3	0.151
Ternera	05/04	569	108	7.88	172.5	<0.1	<0.7	<70	1.25	27.3	<450	2.97	1797		17.0	28.5	0.147
Ternera	05/08	629	66	7.84	181.3	<5	<0.57	<80	1.25	27.8	<190	2.84	1660		16.7	26.0	0.146
Ternera [Galleguillos]	82/05	639	<110	7.80	177.6	<20	<0.3		1.17			2.67	1738		17.7		0.163
Ternera [Galleguillos]	82/10	740	71	7.96	174.9	4	<0.2		1.15			3.07	1614		16.8		0.146
Tinnie	99/02	455	101	7.93	171.4	3.9	0.268	<50	1.02		<200	2.94	1610		16.4	29.3	0.138
Tinnie	99/06	543	100	7.98	175.0	0.62	0.249	<50	0.95		<300	2.90	1632		16.4	30.2	0.132
Tlacotopec	91/05	443	204	7.50	166.5	3.5	<0.273		0.54			2.93	3420		29.8	27.7	0.065
Tlacotopec	92/04	443	189	7.59	166.7	<2.7	0.210		0.42			3.04	3492		30.3	25.1	0.064
Tlacotopec	98/06	448	241	7.79	158.2	4.3	0.520*	<50	0.44		<100	3.18	3320		30.2	27.2	0.063
Tlacotopec	98/04	525	272	7.54	164.2						<100				29.1	27.1	0.063
Warburton Range	77/05	526	104	7.80	182.9	14.7	<1.67		1.03			3.18	1150		12.2		0.165
Warburton Range	78/07	557	51	8.00	175.8	<13	<0.97		1.25			3.32	1367		14.8		0.163
Warburton Range	04/12	524	91	7.71	182.7	5.3	<0.43	<124	1.08	25.8	<150	3.07	1356		14.6	31.2	0.157
Weaver Mountains	91/05	-	115	7.83	176.6	5.5	0.226		0.81			2.78	1570		16.0	25.8	0.122
Weaver Mountains	92/04	425	117	7.77	182.5	<4.7	0.237		0.88			3.08	1655		16.3	31.6	0.120
**SBT**																	
Babb's Mill (Troost's Iron)	83/12	468	79	9.16	175.9	381	17.1		6.01			1.33	2695		32.0		0.992
Babb's Mill (Troost's Iron)	84/03	507	63	9.12	177.6	362	17.8		7.02			1.32	2917		32.3		0.972
Inland Forts 83500	85/10	403	46	9.55	181.1	354	20.1		11.9		138	0.63	477		7.28		1.573
Inland Forts 83500	85/11	442	14	9.73	175.1	336	19.2		11.4			0.81	530		7.10		1.611
South Byron	83/12	347	70	9.22	177.2	383	18.2		6.91			1.21	2458		30.4		1.083
South Byron	84/03	500	63	9.16	175.0	362	17.7		7.72			1.27	2697		30.4		1.157

*Values excluded in the mean calculations.

Table S3. **Modeled bulk compositions of groups IIC, IID, IIF, IVB, and the SBT.** The bold text denotes the optimal bulk compositions.

Group/Trio	P (wt %)	S (wt %)	Fe (mg/g)	Co (mg/g)	Ni (mg/g)	Cu (μg/g)	Ga (μg/g)	Ge (μg/g)	As (μg/g)	Mo (μg/g)	Ru (μg/g)	Rh (μg/g)	Pd (μg/g)	Sb* (ng/g)	W (μg/g)	Re (ng/g)	Os (μg/g)	Ir (μg/g)	Pt (μg/g)	Au (μg/g)
	2.5	4	828.0	5.04	102.0	295	28.5	81.2	16.5	13.8	7.0	1.55	5.5	870	1.70	380	3.8	3.90	8.0	1.45
	2.3	5	821.1	4.90	100.5	310	26.8	75.5	15.3	13.4	6.5	1.50	5.4	840	1.60	350	3.8	3.65	7.6	1.35
**IIC**	**2.2**	**6**	**812.9**	**4.75**	**100.0**	**320**	**25.4**	**71.0**	**15.2**	**13.0**	**5.9**	**1.40**	**5.4**	**860**	**1.50**	**310**	**3.3**	**3.20**	**6.8**	**1.35**
	2.1	7	805.7	4.61	98.7	335	24.0	66.2	14.7	12.8	5.5	1.32	5.4	860	1.35	280	3.3	2.80	6.4	1.30
	1.9	8	798.5	4.49	97.5	355	22.5	61.5	14.1	12.0	5.0	1.25	5.3	860	1.25	250	3.2	2.50	5.8	1.28
	**1.9**	**~0**	**865.6**	**6.80**	**108.0**	**235**	**75.9**	**97.0**	**11.6**		**15.0**		**4.4**	**290**	**3.00**	**1200**	**15.0**	**12.40**	**16.0**	**1.10**
**IID**	1.9	0.5	860.8	6.75	108.0	250	74.8	94.5	11.8		14.6		4.5	310	2.90	1200	14.5	12.30	15.0	1.10
	2.0	1	856.1	6.66	106.8	260	72.0	93.0	12.0		14.2		4.6	350	2.80	1100	14.0	11.50	15.0	1.13
	0.60	4	825.1	6.30	122.0	410	8.0	112.0	11.5	14.0	13.0	1.9	4.9	150	1.00	950	11.0	11.00	17.0	1.05
**IIF**	**0.70**	**5**	**817.2**	**6.15**	**119.0**	**430**	**7.6**	**110.0**	**12.5**	**13.0**	**12.5**	**1.8**	**5.2**	**170**	**0.90**	**830**	**9.5**	**9.00**	**15.0**	**1.10**
	0.65	6	808.9	5.98	118.0	435	7.2	110.0	11.5	13.0	12.0	1.7	5.0	160	0.90	800	9.0	9.00	15.0	1.03
	**0.47**	**~0**	**809.7**	**7.87**	**177.5**	**5**	**0.237**	**0.07**	**1.450**	**34.0**	**26.0**	**3.70**	**9.45**	**8.2**	**3.10**	**2100**	**28.5**	**20.00**	**31.0**	**0.16**
**IVB**	0.45	0.5	804.3	7.82	178.0	5	0.232	0.06	1.418	34.5	25.3	3.58	9.45	8.2	2.95	2050	27.5	19.50	30.0	0.16
	0.43	1	800.8	7.71	177.0	5	0.226	0.06	1.389	34.0	25.0	3.50	9.45	8.2	2.90	2000	26.5	18.50	29.0	0.15
	1.8	6	729.3	8.06	184.0	512	13.2	37.0	18.6	21.5	13.0	3.37	8.7	560	0.46	820	9.0	8.50	15.2	1.83
	1.7	7	724.0	7.86	181.0	525	12.5	34.5	17.9	20.5	12.5	3.16	8.5	565	0.42	690	7.5	7.60	13.9	1.78
**SBT**	**1.5**	**8**	**716.7**	**7.60**	**180.0**	**550**	**11.8**	**32.0**	**17.2**	**20.0**	**11.5**	**2.96**	**8.4**	**580**	**0.40**	**670**	**7.3**	**7.30**	**12.7**	**1.72**
	1.4	9	710.0	7.33	178.0	555	11.0	30.0	16.4	19.5	10.7	2.77	8.4	555	0.36	600	6.6	6.30	11.5	1.64
	1.2	10	705.6	7.12	175.0	590	10.5	28.5	15.7	18.0	9.7	2.60	8.1	550	0.35	550	6.2	5.80	10.7	1.60

*Bulk Sb concentrations of IIC, IID, IIF, IVB, and the SBT are based on two, seven, two, one, and one sample(s), respectively. The estimates of bulk Sb in IIC, IIF, IVB and the SBT may be less reliable than those of other elements.

Table S4. **Summary of bulk HSE and Ni compositions from this study and the literature.** The elements are arranged in order of decreasing volatility.

Group/Trio	Os (µg/g)	Re (ng/g)	Ir (µg/g)	Ru (µg/g)	Pt (µg/g)	Ni (mg/g)	Pd (µg/g)	Reference
IIC	3.35	280	3.05	4.34	6.07	110*	5.30	(*21*)
	3.3	310	3.20	5.9	6.8	100	5.4	This study
IID	-	1100	10.5	-	17.4	105	-	(*22*)
	4.40	370	4.10	6.30	8.0	100	4.30	(*23*)
	15.0	1200	12.4	15.0	16.0	108	4.4	This study
IIF	4.2	355	4.20	6.80	8.20	110*	4.25	(*24*)
	9.5	830	9.00	12.5	15.0	119	5.2	This study
IIIF	3.60	275	3.10	4.30	6.10	80	4.00	(*23*)
IVB	37.0	2800	27.0	27.4	29.5	168	8.6	(*25*)
	21.6	1410	17.5	19.6	29.8	190	10.9	(*27*)
	28.5	2100	20.0	26.0	31.0	178	9.5	This study
SBT	9.40	770	8.50	13.0	16.0	190*	8.40	(*28*)
	7.3	670	7.30	11.5	12.7	180	8.4	This study

*Ni data are from a different study (*23*).

REFERENCES AND NOTES

1. G. Budde, C. Burkhardt, G. A. Brennecka, M. Fischer-Gödde, T. S. Kruijer, T. Kleine, Molybdenum isotopic evidence for the origin of chondrules and a distinct genetic heritage of carbonaceous and noncarbonaceous meteorites. *Earth Planet. Sci. Lett.* **454**, 293–303 (2016).

2. A. Trinquier, J.-L. Birck, C. J. Allegre, Widespread ^{54}Cr heterogeneity in the inner solar system. *Astrophys. J.* **655**, 1179–1185 (2007).

3. A. Trinquier, T. Elliott, D. Ulfbeck, C. Coath, A. N. Krot, M. Bizzarro, Origin of nucleosynthetic isotope heterogeneity in the solar protoplanetary disk. *Science* **324**, 374–376 (2009).

4. C. Burkhardt, T. Kleine, F. Oberli, A. Pack, B. Bourdon, R. Wieler, Molybdenum isotope anomalies in meteorites: Constraints on solar nebula evolution and origin of the Earth. *Earth Planet. Sci. Lett.* **312**, 390–400 (2011).

5. T. S. Kruijer, C. Burkhardt, G. Budde, T. Kleine, Age of Jupiter inferred from the distinct genetics and formation times of meteorites. *Proc. Natl. Acad. Sci.* **114**, 6712–6716 (2017).

6. K. R. Bermingham, E. A. Worsham, R. J. Walker, New insights into Mo and Ru isotope variation in the nebula and terrestrial planet accretionary genetics. *Earth Planet. Sci. Lett.* **487**, 221–229 (2018).

7. J. A. M. Nanne, F. Nimmo, J. N. Cuzzi, T. Kleine, Origin of the noncarbonaceous–carbonaceous meteorite dichotomy. *Earth Planet. Sci. Lett.* **511**, 44–54 (2019).

8. R. N. Clayton, T. K. Mayeda, Oxygen isotope studies of carbonaceous chondrites. *Geochim. Cosmochim. Acta* **63**, 2089–2104 (1999).

9. R. N. Clayton, T. K. Mayeda, J. N. Goswami, E. J. Olsen, Oxygen isotope studies of ordinary chondrites. *Geochim. Cosmochim. Acta* **55**, 2317–2337 (1991).

10. D. S. Grewal, R. Dasgupta, B. Marty, A very early origin of isotopically distinct nitrogen in inner Solar System protoplanets. *Nat. Astron.* **5**, 356–364 (2021).

11. T. S. Kruijer, T. Kleine, L. E. Borg, The great isotopic dichotomy of the early Solar System. *Nat. Astron.* **4**, 32–40 (2020).

12. P. H. Warren, Stable-isotopic anomalies and the accretionary assemblage of the Earth and Mars: A subordinate role for carbonaceous chondrites. *Earth Planet. Sci. Lett.* **311**, 93–100 (2011).

13. E. R. D. Scott, Chemical fractionation in iron meteorites and its interpretation. *Geochim. Cosmochim. Acta* **36**, 1205–1236 (1972).

14. J. T. Wasson, J. Wang, A nonmagmatic origin of group-IIE iron meteorites. *Geochim. Cosmochim. Acta* **50**, 725–732 (1986).

15. J. T. Wasson, *Meteorites: Their Record of Early Solar-System History* (New York: Freeman, 1985).

16. F. Spitzer, C. Burkhardt, F. Nimmo, T. Kleine, Nucleosynthetic Pt isotope anomalies and the Hf-W chronology of core formation in inner and outer solar system planetesimals. *Earth Planet. Sci. Lett.* **576**, 117211 (2021).

17. T. Kleine, K. Mezger, H. Palme, E. Scherer, C. Münker, Early core formation in asteroids and late accretion of chondrite parent bodies: Evidence from 182Hf-182W in CAIs, metal-rich chondrites, and iron meteorites. *Geochim. Cosmochim. Acta* **69**, 5805–5818 (2005).

18. A. Kracher, J. T. Wasson, The role of S in the evolution of the parental cores of the iron meteorites. *Geochim. Cosmochim. Acta* **46**, 2419–2426 (1982).

19. D. J. Malvin, J. H. Jones, M. J. Drake, Experimental investigations of trace element fractionation in iron meteorites. III: Elemental partitioning in the system Fe-Ni-SP. *Geochim. Cosmochim. Acta* **50**, 1221–1231 (1986).

20. J. H. Jones, D. J. Malvin, A nonmetal interaction model for the segregation of trace metals during solidification of Fe-Ni-S, Fe-Ni-P, and Fe-Ni-S-P alloys. *Metall. Mater. Trans. B* **21**, 697–706 (1990).

21. H. A. Tornabene, C. D. Hilton, K. R. Bermingham, R. D. Ash, R. J. Walker, Genetics, age and crystallization history of group IIC iron meteorites. *Geochim. Cosmochim. Acta* **288**, 36–50 (2020).

22. J. T. Wasson, H. Huber, Compositional trends among IID irons; their possible formation from the P-rich lower magma in a two-layer core. *Geochim. Cosmochim. Acta* **70**, 6153–6167 (2006).

23. C. D. Hilton, R. D. Ash, R. J. Walker, Chemical characteristics of iron meteorite parent bodies. *Geochim. Cosmochim. Acta* **318**, 112–125 (2022).

24. C. D. Hilton, R. D. Ash, R. J. Walker, Crystallization histories of the group IIF iron meteorites and Eagle Station pallasites. *Meteorit. Planet. Sci.* **55**, 2570–2586 (2020).

25. A. J. Campbell, M. Humayun, Compositions of group IVB iron meteorites and their parent melt. *Geochim. Cosmochim. Acta* **69**, 4733–4744 (2005).

26. N. L. Chabot, Sulfur contents of the parental metallic cores of magmatic iron meteorites. *Geochim. Cosmochim. Acta* **68**, 3607–3618 (2004).

27. R. J. Walker, W. F. McDonough, J. Honesto, N. L. Chabot, T. J. McCoy, R. D. Ash, J. J. Bellucci, Modeling fractional crystallization of group IVB iron meteorites. *Geochim. Cosmochim. Acta* **72**, 2198–2216 (2008).

28. C. D. Hilton, K. R. Bermingham, R. J. Walker, T. J. McCoy, Genetics, crystallization sequence, and age of the South Byron Trio iron meteorites: New insights to carbonaceous chondrite (CC) type parent bodies. *Geochim. Cosmochim. Acta* **251**, 217–228 (2019).

29. H. A. Tornabene, "Insights to the Genetics, Age and Crystallization of Group IC and IIC Iron Meteorites", thesis, University of Maryland, College Park (2020).

30. J. T. Wasson, H. Huber, D. J. Malvin, Formation of IIAB iron meteorites. *Geochim. Cosmochim. Acta* **71**, 760–781 (2007).

31. J. T. Wasson, Trapped melt in IIIAB irons; solid/liquid elemental partitioning during the fractionation of the IIIAB magma. *Geochim. Cosmochim. Acta* **63**, 2875–2889 (1999).

32. N. L. Chabot, B. Zhang, A revised trapped melt model for iron meteorites applied to the IIIAB group. *Meteorit. Planet. Sci.* **57**, 200–227 (2022).

33. J. T. Wasson, J. W. Richardson, Fractionation trends among IVA iron meteorites: Contrasts with IIIAB trends. *Geochim. Cosmochim. Acta* **65**, 951–970 (2001).

34. T. J. McCoy, R. J. Walker, J. I. Goldstein, J. Yang, W. F. McDonough, D. Rumble, N. L. Chabot, R. D. Ash, C. M. Corrigan, J. R. Michael, P. G. Kotula, Group IVA irons: New constraints on the crystallization and cooling history of an asteroidal core with a complex history. *Geochim. Cosmochim. Acta* **75**, 6821–6843 (2011).

35. A. E. Rubin, B. Zhang, N. L. Chabot, IVA iron meteorites as late-stage crystallization products affected by multiple collisional events. *Geochim. Cosmochim. Acta* **331**, 1–17 (2022).

36. B. Zhang, N. L. Chabot, A. E. Rubin, M. Humayun, J. S. Boesenberg, D. van Niekerk, Chemical study of group IIIF iron meteorites and the potentially related pallasites Zinder and Northwest Africa 1911. *Geochim. Cosmochim. Acta* **323**, 202–219 (2022).

37. N. L. Chabot, E. A. Wollack, W. F. McDonough, R. D. Ash, S. A. Saslow, Experimental determination of partitioning in the Fe-Ni system for applications to modeling meteoritic metals. *Meteorit. Planet. Sci.* **52**, 1133–1145 (2017).

38. N. L. Chabot, J. H. Jones, The parameterization of solid metal-liquid metal partitioning of siderophile elements. *Meteorit. Planet. Sci.* **38**, 1425–1436 (2003).

39. J. H. Jones, M. J. Drake, Experimental investigations of trace element fractionation in iron meteorites, II: The influence of sulfur. *Geochim. Cosmochim. Acta* **47**, 1199–1209 (1983).

40. J. Willis, J. I. Goldstein, The effects of C, P, and S on trace element partitioning during solidification in Fe-Ni alloys. *J. Geophys. Res. Solid Earth* **87**, A435–A445 (1982).

41. J. T. Wasson, The chemical classification of iron meteorites—III. Hexahedrites and other irons with germanium concentrations between 80 and 200 ppm. *Geochim. Cosmochim. Acta* **33**, 859–876 (1969).

42. J. T. Wasson, B.-G. Choi, E. A. Jerde, F. Ulff-Møller, Chemical classification of iron meteorites: XII. New Members of the Magmatic Groups. *Geochim. Cosmochim. Acta* **62**, 715–724 (1998).

43. T. J. McCoy, C. M. Corrigan, K. Nagashima, V. S. Reynolds, R. D. Ash, W. F. McDonough, J. Yang, J. I. Goldstein, C. D. Hilton, The Milton pallasite and South Byron Trio irons: Evidence for oxidation and core crystallization. *Geochim. Cosmochim. Acta* **259**, 358–370 (2019).

44. B. J. Wood, D. J. Smythe, T. Harrison, The condensation temperatures of the elements: A reappraisal. *Am. Mineral.* **104**, 844–856 (2019).

45. H. Wänke, H. Baddenhausen, H. Palme, B. Spettel, On the chemistry of the Allende inclusions and their origin as high temperature condensates. *Earth Planet. Sci. Lett.* **23**, 1–7 (1974).

46. M. Pan, Kinetic condensation of metals in the early solar system: Unveiling the cooling history of solar nebula by refractory metal nuggets. *Icarus* **350**, 113851 (2020).

47. A. E. Rubin, Carbonaceous and noncarbonaceous iron meteorites: Differences in chemical, physical, and collective properties. *Meteorit. Planet. Sci.* **53**, 2357–2371 (2018).

48. J. T. Wasson, G. W. Kallemeyn, Compositions of chondrites. *Philos. Trans. Royal Soc. A* **325**, 535–544 (1988).

49. A. E. Rubin, Origin of the differences in refractory-lithophile-element abundances among chondrite groups. *Icarus* **213**, 547–558 (2011).

50. D. Wark, W. V. Boynton, The formation of rims on calcium-aluminum-rich inclusions: Step I—Flash heating. *Meteorit. Planet. Sci.* **36**, 1135–1166 (2001).

51. G. J. Consolmagno, D. T. Britt, R. J. Macke, The significance of meteorite density and porosity. *Geochemistry* **68**, 1–29 (2008).

52. J. M. Sunshine, H. V. Connolly Jr., T. J. McCoy, S. J. Bus, L. M. La Croix, Ancient asteroids enriched in refractory inclusions. *Science* **320**, 514–517 (2008).

53. F. Spitzer, C. Burkhardt, G. Budde, T. S. Kruijer, A. Morbidelli, T. Kleine, Isotopic evolution of the inner solar system inferred from molybdenum isotopes in meteorites. *Astrophys. J. Lett.* **898**, L2 (2020).

54. G. A. Brennecka, C. Burkhardt, G. Budde, T. S. Kruijer, F. Nimmo, T. Kleine, Astronomical context of Solar System formation from molybdenum isotopes in meteorite inclusions. *Science* **370**, 837–840 (2020).

55. T. Yokoyama, Y. Nagai, R. Fukai, T. Hirata, Origin and evolution of distinct molybdenum isotopic variabilities within carbonaceous and noncarbonaceous reservoirs. *Astrophys. J.* **883**, 62 (2019).

56. M. Fischer-Göedde, D. Schwander, U. Ott, Ruthenium isotope composition of allende refractory metal nuggets. *AJ* **156**, 176–187 (2018).

57. T. S. Kruijer, M. Touboul, M. Fischer-Gödde, K. Bermingham, R. J. Walker, T. Kleine, Protracted core formation and rapid accretion of protoplanets. *Science* **344**, 1150–1154 (2014).

58. A. M. Davis, Volatile evolution and loss, in *Meteorites and the Early Solar System II*, D. S. Lauretta, H. Y. McSween Jr., Eds. (University of Arizona Press, Tucson, 2006) vol. 1, pp. 295–307.

59. M. M. Hirschmann, E. A. Bergin, G. A. Blake, F. J. Ciesla, J. Li, Early volatile depletion on planetesimals inferred from C–S systematics of iron meteorite parent bodies. *Proc. Natl. Acad. Sci.* **118**, e2026779118 (2021).

60. T. Kleine, M. Matthes, F. Nimmo, I. Leya, Silver isotopic evidence for impact-driven volatile loss from differentiated asteroids, in *49th Lunar and Planetary Science Conference* (2018), pp. 1963.

61. J. T. Wasson, W.-H. Choe, The IIG iron meteorites: Probable formation in the IIAB core. *Geochim. Cosmochim. Acta* **73**, 4879–4890 (2009).

62. N. L. Chabot, Composition of metallic cores in the early Solar System, in *49th Lunar and Planetary Science Conference* (2018), pp. 1532.

63. S. J. Desch, A. Kalyaan, C. M. O'D. Alexander, The effect of Jupiter's formation on the distribution of refractory elements and inclusions in meteorites. *Astrophys. J., Suppl. Ser.* **238**, 11 (2018).

64. E. R. D. Scott, A. N. Krot, Chondrites and their components, in *Meteorites and Cosmochemical Processes, Treatise on Geochemistry*, A. M. Davis, H. D. Holland, K. K. Turekian, Eds. (Elsevier, 2003), vol. 1, pp. 711.

65. A. Kracher, J. Willis, J. T. Wasson, Chemical classification of iron meteorites—IX. A new group (IIF), revision of IAB and IIICD, and data on 57 additional irons. *Geochim. Cosmochim. Acta* **44**, 773–787 (1980).

66. E. R. D. Scott, J. T. Wasson, Chemical classification of iron meteorites—VIII. Groups IC. IIE, IIIF and 97 other irons. *Geochim. Cosmochim. Acta* **40**, 103–108 (1976).

67. R. Schaudy, J. T. Wasson, V. F. Buchwald, The chemical classification of iron meteorites. VI. A reinvestigation of irons with Ge concentration lower than 1 ppm. *Icarus* **17**, 174–192 (1972).

68. J. T. Wasson, X. Ouyang, J. Wang, J. Eric, Chemical classification of iron meteorites: XI. Multi-element studies of 38 new irons and the high abundance of ungrouped irons from Antarctica. *Geochim. Cosmochim. Acta* **53**, 735–744 (1989).

69. W. F. McDonough, S.-S. Sun, The composition of the Earth. *Chem. Geol.* **120**, 223–253 (1995).

70. V. F. Buchwald, *Handbook of Iron Meteorites: Their History, Distribution, Composition and Structure* (Arizona State University, 1975).

71. J.T. Wasson, Vesta and extensively melted asteroids: Why HED meteorites are probably not from Vesta. *Earth Planet. Sci. Lett.* **381,** 138–146 (2013).
